# Small groups and long memories promote cooperation

**DOI:** 10.1038/srep26889

**Published:** 2016-06-01

**Authors:** Alexander J. Stewart, Joshua B. Plotkin

**Affiliations:** 1 Department of Biology, University of Pennsylvania, Philadelphia, PA 19104, USA

## Abstract

Complex social behaviors lie at the heart of many of the challenges facing evolutionary biology, sociology, economics, and beyond. For evolutionary biologists the question is often how group behaviors such as collective action, or decision making that accounts for memories of past experience, can emerge and persist in an evolving system. Evolutionary game theory provides a framework for formalizing these questions and admitting them to rigorous study. Here we develop such a framework to study the evolution of sustained collective action in multi-player public-goods games, in which players have arbitrarily long memories of prior rounds of play and can react to their experience in an arbitrary way. We construct a coordinate system for memory-*m* strategies in iterated *n*-player games that permits us to characterize all cooperative strategies that resist invasion by any mutant strategy, and stabilize cooperative behavior. We show that, especially when groups are small, longer-memory strategies make cooperation easier to evolve, by increasing the number of ways to stabilize cooperation. We also explore the co-evolution of behavior and memory. We find that even when memory has a cost, longer-memory strategies often evolve, which in turn drives the evolution of cooperation, even when the benefits for cooperation are low.

Behavioral complexity is a pervasive feature of organisms that engage in social interactions. Rather then making the same choices all the time – always cooperate, or never cooperate – organisms behave differently depending on their social environment or their past experience. The need to understand behavioral complexity is at the heart of many important challenges facing evolutionary biology as well as the social sciences, or indeed any problem in which social interactions play a part. Cooperative social interactions in particular play a central role in many of the major evolutionary transitions, from the emergence of multi-cellular life to the development of human language^1^.

Evolutionary biologists have been successful in pinpointing biological and environmental factors that influence the emergence of cooperation in a population. The demographic and spatial structure of populations in particular have emerged as fundamentally important factors^2^,^3^,^4^,^5^,^6^,^7^,^8^. At the other end of the scale, the underlying mechanisms of cooperation – such as the genetic architectures that encode social traits, or the ability of public goods to diffuse in the environment – also place constraints on how and to what extent cooperation will evolve^9^,^10^,^11^,^12^,^13^.

Despite extensive progress for simple interactions, an understanding of the evolution of cooperation when social interactions occur repeatedly – so that individuals can update their behavior in the light of past experience – and involve multiple participants simultaneously, remains elusive. Some of the most promising approaches for tackling this problem come from the study of iterated games^14^,^15^,^16^,^17^,^18^,^19^,^20^,^21^,^22^,^23^. In the language of game theory, behavioural updates in light of past experience are modelled as a strategy in an iterated multi-player game among heterogenous individuals. Even when we limit ourselves to a small set of relatively simple strategies in such games, the resulting evolutionary dynamics are often surprising and counter-intuitive. As we begin to allow for a wider array of ever more complex behaviors, results on the emergence of cooperation are correspondingly harder to pin down.

Nowhere is the complicated nature of the problem more evident than in the discussion surrounding the role of memory in iterated games and the evolution of cooperation^22^,^23^,^24^,^25^. On the one hand, memory can obviously be a powerful force for promoting cooperation if it allows players to recognize kin, or to otherwise tag different opponents^26^,^27^. Yet, such recognition is likely to be costly and complex to evolve^23^,^28^,^29^. On the other hand, a simpler form of memory that is confined to past interactions within the course of a given iterated game, so that a player’s strategy can take into account multiple rounds of past play, might be relatively easy to evolve and incur fewer costs^24^,^25^,^29^. Nevertheless, the usefulness of such memory within the course of an iterated game has been called into question by the results of Press and Dyson^18^ and their generalization for multi-player games^30^, which show that a player with memory-1 can treat all of her opponents as though they too use a memory-1 strategy, regardless of the opponent’s actual memory capacity. Thus a memory-1 strategy that stabalizes cooperation against any memory-1 invader also stabalizes cooperation against any arbitrary invader^19^,^31^,^32^,^33^,^34^.

If we are interested in the evolution of cooperation, however, the stability of some memory-1 strategies against all forms of invaders is not in itself informative. Rather, as we and others have shown, what matters most for the maintenance of cooperation over evolutionary time is the ease with which successful cooperative strategies can evolve^31^,^34^,^35^. If the ability to recall past interactions makes cooperative strategies easier or harder to evolve, then memory can facilitate or impede the evolution of cooperation. The central question of this paper then, is: what effect does the ability to recall prior rounds of play have on the evolution of successful cooperative strategies in iterated multi-player games?

We study evolving populations composed of individuals playing arbitrary strategies in infinitely iterated, multiplayer games. We focus on the prospects for cooperation in public goods games, and we investigate how these prospects depend on the number of players that simultaneously participate in the game, on the memory capacity of the players, and on the total population size. We then study the co-evolution of players’ strategies alongside their capacity to remember prior interactions, including when such memory capacity comes at a cost. We arrive at a simple insight: when games involve few players and groups are small, longer memory strategies tend to evolve, which in turn increases the amount of cooperation that can persist. And so populations tend to progress from short memories and selfish behavior to long memories and cooperation.

## Methods

**Figure 1:**
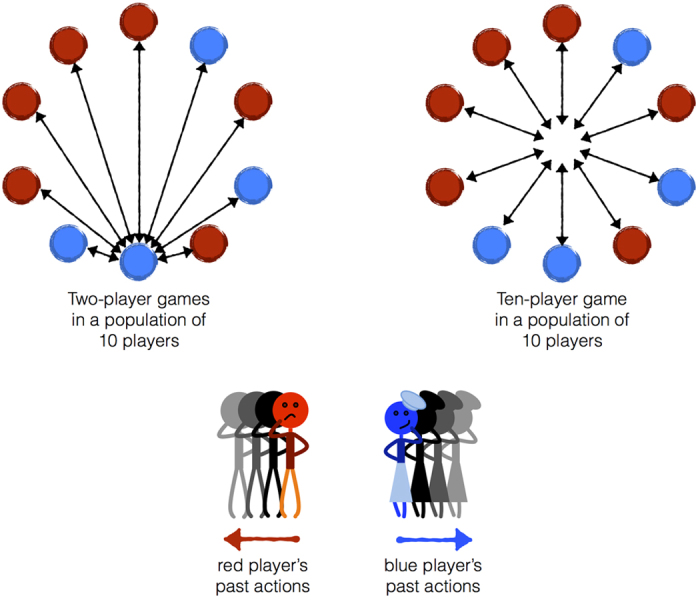
Multiplayer games and memory. We study the evolution of behavior in iterated *n*-player public-goods games in which players use strategies with memory capacity *m*. We consider a replicating population of *N* individuals who each receive a payoff from engaging in an infinitely iterated game with all possible subsets of (*n* − 1) opponents in the population. Players then reproduce according to a “copying process”, in which a player *X* copies another player’s strategy *Y* with a probability 
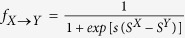
 where *S*^*X*^ and *S*^*Y*^ are the player’s respective payoffs and *s* scales the strength of selection. We consider the case of strong selection, such that a rare mutant who is at a selective disadvantage is quickly lost from the population^31^. We investigate the success of cooperative strategies as a function of group size and the length of players’ memories. We determine the frequency of robust cooperative strategies, which can resist invasion by any possible mutant. (Top) Depending on the size of the game *n* relative to the population *N*, the dynamics of public-goods games are different. In a two-player game, a series of pairwise interactions occur in the population at each generation (left). If the whole population plays the game each generation (right) all players interact simultaneously. (Bottom) Memory of past events results in strategies that update behavior depending on the histories of both players’ actions. This allows for more complex strategies, such as those that punish rare defection or reward rare cooperation.

We study the evolution of cooperation in iterated public-goods games, in which *n* players repeatedly choose whether to cooperate by contributing a cost *C* to a public pool, producing a public benefit *B* > *C*. In each round of iterated play the total benefit produced due to all player’s contributions is divided equally between all players. Thus, if *k* players choose to cooperate in a given round, each player receives a benefit *Bk*/*n*. We study finite populations of *N* players engaging in infinitely iterated *n*-player public-goods games, using strategies with memory length *m*, meaning a player can remember how many times she and her opponents cooperated across the preceding *m* rounds (Fig. 1).

We focus on the evolution of sustained collective action, meaning the evolution of strategies that, when used by each member of the population, produce an equilibrium play with all players cooperating each round. This may be thought of as the best possible social outcome of the game, because it produces the maximum total public good. We contrast the prospects for sustained cooperation with the prospects for sustained *inaction*, meaning strategies that, when used by each member of the population, produce an equilibrium play with all players defecting each round. This may be thought of as the worst possible social outcome of the game, because it results in no public good being produced at all.

To study the evolutionary prospects of collective action and inaction we determine the “volume of robust strategies” that produce sustained cooperation or defection in a repeated *n*-player game, in which players have memory *m*. The game is played in a well-mixed population, composed of *N* haploid individuals who reproduce according to a “copying process” based on their payoffs (Fig. 1)^36^. The volume of robust strategies measures how much cooperation or defection will evolve across many generations^31^. More specifically, this volume is the probability that a randomly drawn strategy that produces sustained cooperation (or defection) can resist invasion by all other possible strategies that do not produce sustained cooperation (or defection)^19^,^31^,^32^,^33^,^34^. As we have shown previously^31^, the volumes of robust strategies determine the evolutionary dynamics of cooperation and defection in iterated games. We confirm the utility of this approach by comparing our analytical predictions to Monte Carlo simulations, studying the effects of population size, group size, and memory capacity on the evolution of cooperation.

We begin our analysis by describing a coordinate system under which the volume of robust strategies can be determined analytically, for games of size *n*, played in populations of size *N*, in which strategies have memory length *m*. We use this coordinate system to completely characterize all evolutionary robust cooperating (and defecting) strategies, which cannot be invaded by any non-cooperating (or non-defecting) mutants, in the iterated *n*-player public-goods game. We apply these results to make specific predictions for the effects of group size and of memory capacity on the evolution of collective action through sustained cooperation. Finally we explore the consequences of these predictions for the co-evolution of cooperation and memory capacity itself.

### Beyond two-player games and memory-1 strategies

Recently, Press and Dyson introduced so-called zero determinant (ZD) strategies in iterated two-player games^18^. ZD strategies are of interest because, when a player unilaterally adopts such a strategy she enforces a linear relationship between her longterm payoff and that of her opponent, and thereby gains some measure of control over the outcome of the game^37^,^38^,^39^,^40^,^41^. Several authors have worked to extend the framework of Press and Dyson to multi-player games^30^,^39^ and have characterized multi-player ZD strategies, revealing a number of interesting properties.

Other research has expanded the framework of Press and Dyson to study all possible memory-1 strategies for infinitely repeated, two-player games^19^,^31^,^32^,^33^,^34^. This work involves developing a coordinate system for the space of all memory-1 strategies^19^ that allows us to describe a straightforward (although not necessarily linear) relationship between the two players’ longterm payoffs. This relationship between players’ longterm payoffs, in turn, has enabled us to fully characterize all memory-1 Nash equilibria and all evolutionary robust strategies for infinitely repeated two-player games, played in a replicating population of *N* individuals^19^,^31^,^32^,^34^.

Here we generalize this body of work by developing a coordinate system for the space of memory-*m* strategies in multi-player games of size *n*, such that all *n* players’ longterm payoffs are related in a straightforward (although not necessarily linear) way. One essential trick that enables us to achieve this goal is to construct a mapping between memory-*m* strategies in an *n*-player game and memory-1 strategies in an associated *n* × *m*-player game. We then construct a coordinate system for the space of memory-1 strategies in multi-player games that allows us to easily characterize the cooperating and the defecting strategies that resist invasion. We apply these techniques to the case of iterated *n*-player public-goods games and we precisely characterize all evolutionary robust memory-*m* strategies – i.e. those strategies that, when resident in a finite population of *N* players, can resist selective invasion by all other possible strategies – thereby elucidating the prospects for the evolution of cooperation in a very general setting.

### A coordinate system for long-memory strategies in multi-player games

Our goal is to study the effects of group size and memory on the frequency and nature of collective action in public-goods games. Allowing for long-memory strategies and games with more than two players greatly expands the potential for behavioral complexity, because players are able to react to the behaviors of multiple opponents across multiple prior interactions. And so merely determining the payoffs received by players in such an iterated public-goods game can pose a significant challenge. In order to tackle this problem we develop a coordinate system for parameterizing strategies, in which the outcome of a game between multiple players using long-memory strategies can nonetheless be easily understood.

A player using a memory-*m* strategy chooses her play in each round of an iterated game in a way that depends on the history of plays by all *n* players across the preceding *m* rounds. In general such a strategy consists of 2^*n*×*m*^ probabilities for cooperation in the present round. We write the probability for cooperation of a focal player in its most general form as 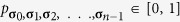 where 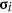 denotes the history of plays for player *i*. Each 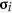 corresponds to an ordered sequence of *m* plays for player *i*, with each entry taking the value *c* (cooperate) or *d* (defect). The 2^*n*×*m*^ probabilities for cooperation form a basis for 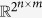 and constitute a system of coordinates for the space of memory-*m* strategies in *n*-player games. In the supporting information we describe in detail how to construct an alternate coordinate system of 2^*n*×*m*^ vectors that also form a basis for 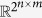, and which greatly simplifies the analysis of long-term payoffs in iterated games. Below we describe this alternative coordinate system for the specific case of iterated public-goods games, which are the focus of this study.

#### (i) Mapping memory-*m* to memory-1

In order to simplify our analysis of long-memory strategies we will conceive of a focal player using a memory-*m* strategy in an *n*-player game as a player who instead uses a memory-1 strategy in an associated *n* × *m*-player game. That is, we will think of an *n*-player game in which a focal player uses a memory-*m* strategy in terms of an equivalent *n* × *m*-player game, which is composed of *n* “real” players along with *m* − 1 “shadow” players associated with each real player. The shadow players play the same way that their associated real player did *t* rounds previously, for 2 ≤ *t *≤ *m*. The focal player’s memory-*m* strategy is thus identical to a memory-1 strategy in the *n* × *m* player game, where the corresponding memory-1 strategy responds to a large set of “shadow” players whose actions in the immediately previous round simply encode the actions taken by the *n* real players in the preceding *m* rounds. This trick allows us to reduce the problem of studying long-memory strategies to the problem of studying memory-1 strategies, albeit with a larger number of players in the game.

All that is required is to construct strategies for the shadow players so that the state of the system across the preceding *m* rounds is correctly recreated at each round of the associated *n *× *m*-player game. This construction is straight forward. If the focal player played *c* in the last round, then we stipulate that her first shadow player will play *c* in the next round (i.e. it will copy her last move). Similarly her second shadow player will copy the last move of her first shadow player, and so on, up to her (*m* − 1)st shadow player. The same goes for the shadow players of each of her *n *− 1 opponents. In this way, all the plays of the last *m* rounds in the *n*-player game are encoded at each round in the associated *n *× *m*-player game.

Having transformed an arbitrary memory-*m* strategy in an *n*-player into an associated memory-1 strategy in an *n *× *m*-player game, we now describe a coordinate system for memory-1 strategies that allows us to derive a simple relation among the equilibrium payoffs to all players. We define this coordinate system for arbitrary games in the supporting information (section 3), and for the case of public-goods games below.

#### (ii) Parameterizing strategies in public-goods games

Under a public-goods game, a player who cooperates along with *k* of her opponents receives a net payoff *B*(*k* + 1)/*n* − *C*, whereas a player who defects while *k* of her opponents cooperate receives a net payoff *Bk*/*n*. That is, the payoff received depends on whether or not the focal player cooperated and on the number of her opponents that cooperated, but it does not depend on the identity of her cooperating opponents. Likewise, if a player has memory of the preceding *m* rounds of an iterated public-goods game, then her payoff across those rounds depends on the total number of times she cooperated and the total number of times her opponents cooperated, but it does not depend on the order in which different players cooperated nor on the identity of her cooperating opponents. Therefore, rather than studying the full space of 2^*n*×*m*^ probabilities for cooperation, we can limit our analysis for iterated public-goods games to strategies that keep track of the total number of times a focal player cooperated, and the number of times her opponents cooperated, within her memory capacity. A focal player’s strategy can thus be expressed as 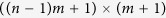 probabilities for cooperation each round, 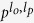, where *l*_*o*_ denotes the total of number of times the player’s opponents cooperated in the preceding *m* rounds (which number can vary between 0 and (*n* − 1)*m*) and *l*_*p*_ denotes the total number of times the player herself cooperated in the preceding *m* rounds (which can vary between 0 and *m*).

**Figure 2:**
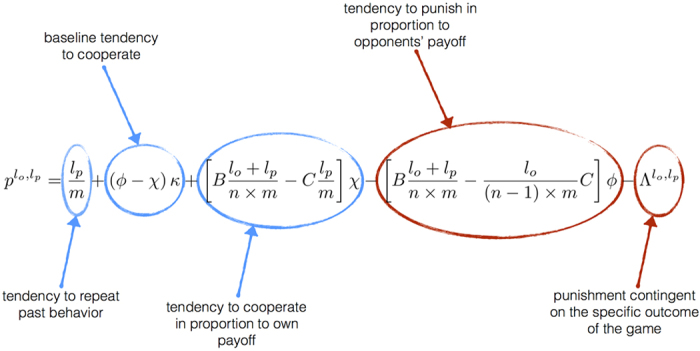
A co-ordinate system for describing strategies in public-goods games. We consider the space of strategies of the form 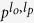, such that players cooperate with a probability that depends on the number of times *l*_*o*_ her opponents have cooperated and the number of times *l*_*p*_ she has cooperated within her memory. We define the strategy of a focal player by coordinates 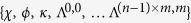 as shown in the figure. The components of this coordinate system have an intuitive interpretation: the probability that a player cooperates depends on (1) her past tendency to cooperate, (2) a baseline tendency to cooperate (*κ*), (3) a tendency to cooperate in proportion to her own payoff (*χ*), (4) a tendency to punish (i.e. defect) in proportion to her opponents’ payoffs (*ϕ*) and (5) a contingent punishment that depends on the specific outcome of the game over the prior *m* rounds (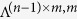).

Although the probabilities 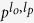 are perhaps the most natural coordinates for describing a memory-*m* strategy in an iterated *n*-player public-goods game, we have developed an alternative coordinate system, defined in Fig. 2, that simplifies the analysis of equilibrium payoffs and the evolutionary robustness of strategies. The alternative system of 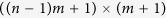 coordinates for a given player’s strategy is described by parameters 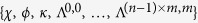 defined in Fig. 2. We impose the boundary conditions 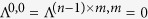 along with one other linear relationship on the Λ terms (see supporting information). Qualitatively, this coordinate system describes the probability of cooperation in a given round, 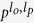, in terms of a weighted sum of five components: (1) The tendency to repeat past behavior; (2) The baseline tendency to cooperate (*κ*); (3) The tendency to cooperate in proportion to the payoff received by the focal player (χ); (4) The tendency to punish (i.e. defect) in proportion to the payoffs received by her opponents (*φ*) and (5) The tendency to punish in response to the specific outcome of the previous rounds (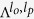).

The advantage of using this coordinate system is that it provides a simple relationship between the long-term payoff to a focal player 0, *S*^0^, and the the long-term payoffs *S*^*i*^ of each of her opponents *i* in an iterated *n*-player public-goods game:


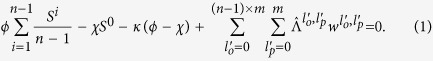


Here the term 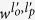 denotes the equilibrium rate at which the invading player cooperates 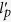 times and his opponents cooperate 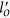 times over the preceding *m* rounds, and 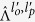 denotes the contingent punishment of the focal strategy from the point of view of a co-player (see supporting information for a derivation of 1). 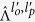 is related in a simple way to the terms 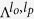, so that increasing 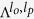 increases 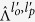 (see supporting information).

## Results

### The effects of group size on robust cooperation

The relationship among payoffs summarized in 1 provides extensive insight into the outcome of iterated public-goods games. Of particular interest are the prospects for cooperation as the group size *n* and population size *N* grow. Public-goods games are well known examples of the collective action problem, in which increasing the number of players in a game worsens the prospects for cooperation^42^,^43^. Larger populations, on the other hand, tend to make it easier to evolve robust cooperation, at least for two-player games^32^. We will use 1 to explore the tradeoff between group size and population size, and the nature of robust cooperative behaviors that can evolve in multi-player games.

1 allows us to characterize the ability of a cooperative strategy to resist invasion by any other strategy in a population of size *N*^19^,^31^,^32^,^34^. We define a cooperative strategy as one which, when played by every member of a population, assures that all players cooperate at equilibrium and thus receive the payoff for mutual cooperation, *B*–*C*. This implies the necessary condition 
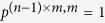
, so that if all players cooperated in the preceding *m* rounds, a player using a cooperative strategy is guaranteed to cooperate in the next round. We call such strategies “cooperators” meaning that they produce sustained cooperation when resident in a population. In the alternate coordinate system developed above a necessary condition for sustained cooperation is *κ* = *B*–*C*.

Conversely, we also consider strategies that lead to collective *inaction*, meaning sustained defection. Such strategies must have 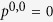, which implies a necessary condition *κ* = 0 in the alternate coordinate system. We call strategies satisfying this condition “defectors” meaning that they produce sustained defection when resident in a population.

A rare mutant *i* can invade a population of size *N* in which a cooperative strategy is resident only if he receives a payoff *S*^*i*^ that exceeds the payoff received by the resident cooperator. By considering bounds on the payoffs received by players (see supporting information) we have derived necessary and sufficient conditions for a cooperative strategy 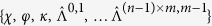 to resist selective invasion by any mutant strategy – that is, for a cooperative strategy to be evolutionary robust:


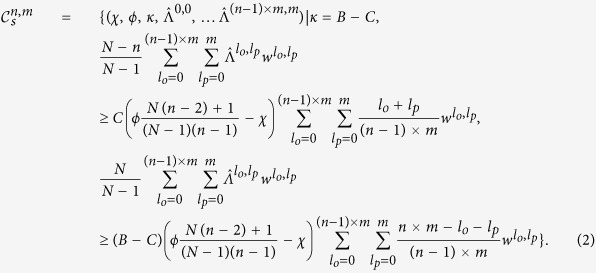


Eq. 2 allows us to make a number of observations about the prospects and nature of robust cooperation. First, although Eq. 2 depends on the equilibrium rate of play 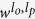, which itself depends on the strategy used by both the focal strategy and the co-player, because 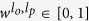 we can nonetheless choose 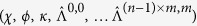 so as to ensure Eq. 3 is satisfied and the strategy is robust against all mutants. In particular, for a given *ϕ*,*χ* if we choose large enough values of 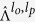 the strategy will be robust. This corresponds to ensuring contingent punishment is “strong enough” so that players are able to successfully punish rare defection. Second, positive values of *χ*, corresponding to more generous strategies^32^, in which players tend to share the benefits of mutual cooperation, also make it easier for a strategy to satisfy the requirements for robust cooperation. Thus, complex strategies that punish rare defection and are generous to other players tend to produce robust cooperative behavior in an evolving population. Finally, we can use Eq. 2 to assess the robustness of *any* cooperative strategy, by calculating the equilibrium rate of play against four “extremal” strategies that maximize or minimize the sum and the difference of the players’ scores (see SI and also^39^).

Eq. 2 also shows that larger values of *n*, corresponding to games with more players, tend to make for smaller volumes of robust cooperative strategies. This can be see on the left-hand side of the first inequality in 2, where increasing *n* attenuates the impact of contingent punishment on robustness. Likewise, this can also been seen on the right-hand side of the inequality in 2, where increasing *n* attenuates the impact of generosity on robustness.

The effects of group size (i.e the number of players in a given game) on the prospects for cooperation can be illustrated by considering two extreme cases. When the entire population takes part in a single multi-player game, so that *n *= *N*, then 2 implies that strategies can be robust only if *χ* ≥ *ϕ*. However, in order to produce a viable strategy *χ* ≤ *ϕ* is required (Fig. 2); and so the only possible way to ensure robust cooperation in this extreme case is to have *χ* = *ϕ*. The condition *χ* = *ϕ* gives a tit-for-tat-like strategy, and it results in unstable cooperative behavior in the presence of noise^31^. And so, in the limit of games as large as the entire population size the prospects for evolutionary robust cooperation are slim. However, in the contrasting case in which the population size is much larger than the size of the game being played, that is 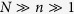, then 2 shows that a positive volume of robust cooperative strategies always exists, given sufficient contingent punishment 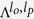, even in very large games.

Understanding the expected rate of cooperation in multi-player games requires that we compare the volume of robust cooperative strategies to the volume of robust defecting strategies. A rare mutant *i* can invade a population in which a defecting strategy is resident only if he receives a payoff *S*^*i*^ that exceeds the payoff received by the resident defector. The resulting necessary and sufficient conditions for the robustness of defecting strategies are then:


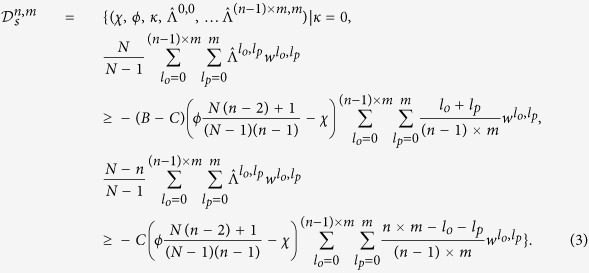


Once again, we see from Eq. 3 that if we choose large enough values of 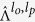, resulting in stronger contingent punishment of rare cooperators in a population of defectors, a defecting strategy will be robust. However, in contrast to the case for cooperators, *smaller* values of *χ*, which for defectors corresponds to more extortionate behavior, such that players try to increase their own payoff at their opponents’ expense^18^, makes a defecting strategy more likely to satisfy the requirements for robustness. Finally, while larger values of *n* can attenuate the effect of contingent punishment on robustness, they also make it easier to construct an extortionate strategy that is robust; and the latter effect is always stronger, so that larger games permit a greater volume of defecting strategies. Overall, Eq. 3 implies that increasing the number of players in each game, *n*, tends to increase the volume of robust defectors, in contrast to its effect on robust cooperators.

**Figure 3:**
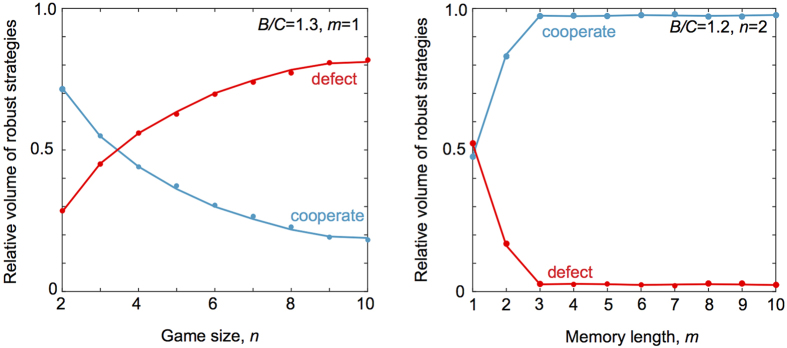
The impact of group size and memory capacity on cooperation. We calculated the relative volumes of robust cooperation – that is, the absolute volume of robust cooperative strategies divided by the total volume of robust cooperators and defectors – and compared this to the relative volume of defectors (solid lines) by numerically integrating Eqs. 2, 3. We also verified these analytic results by randomly drawing 10^6^ cooperative and 10^6^ defecting strategies and determining their success at resisting invasion from 10^5^ random mutants with the same memory (points). We calculated player’s payoffs by simulating 2 × 10^3^ rounds of a public-goods game. We then plotted the relative volumes of robust cooperators and robust defectors as a function of group size *n* (with fixed memory *m* = 1, left) and as a function of memory capacity *m* (with fixed group size *n *= 2, right). Increasing group size increases the relative volume of robust defection; while increasing memory length increases the relative volume of robust cooperation. In all calculations and simulations we used cost *C* = 1 and benefit *B* as indicated in the figure.

We confirmed our predictions for the effects of group size on the volume of robust cooperators and defectors by analytical calculation of robust volumes, from Eqs 2, 3, and by comparison to direct simulation for the invasibility of cooperators and defectors against a large range of mutant invaders (Fig. 3a). As group size increases the volume of robust cooperators decreases relative to the volume of robust defectors, making cooperation harder to evolve.

There is a simple intuition for why larger games make cooperation less robust and defection more robust: In public-goods games with more players, the marginal change in payoff to a player who switches from cooperation to defection is *C* − *B*/*n*, and so the incentive to defect grows as the size of the group grows. This of course is the group size paradox, and it is a well known phenomenon for any collective action problem^42^. In the limiting case *n* = *N* the only hope for robust cooperation is tit-for-tat-like strategies, that are capable of both sustained cooperation and sustained defection, depending on their opponent’s behavior.

In general, both cooperators and defectors have positive volumes of robust strategies, provided *n* < *N*. As such, both cooperation and defection can evolve. Although these robust strategies cannot be selectively invaded by any other strategy when resident in a population, they can be neutrally replaced by a non-robust strategy of the same type, which can in turn be selectively invaded. As a result, there is a constant turnover between cooperation and defection over the course of evolution, with the relative time spent at cooperation versus defection determined by their relative volumes of robust strategies^31^,^34^.

Our results show that the problem of collective action is alleviated by sufficiently large population sizes. That is, for an arbitrarily large group size *n* we can always find yet larger population sizes *N* such that robust cooperative strategies are guaranteed to exist. Moreover, increasing the population size *N* leads to increasing volumes of robust cooperative strategies and decreasing volumes of robust defecting strategies (Fig. S1).

### The effects of memory on robust cooperation

We have not yet said anything about the impact of memory capacity on the prospects for cooperation. Indeed, the robustness conditions Eqs 2, 3 do not depend explicitly on memory length *m*, as they do on group size *n* and on population size *N*. However, memory does have an important impact on the efficacy of contingent punishment, 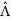, on the left-hand sides of the inequalities in 2 and 3. Figure 3 illustrates the impact of increasing memory *m* on the volume of robust cooperative and robust defecting strategies. Here we see the opposite pattern to the effect for group size: as memory increases, there is a larger volume of robust cooperation relative to robust defection.

We can develop an intuitive understanding for the effect of memory on sustained cooperation by considering its role in producing effective punishment. A longer memory enables a player to punish opponents who seek to gain an advantage through rare deviations from the social norm: that is, rare defectors in a population of cooperators or rare cooperators in a population of defectors. However, using a long memory to punish rare defectors is a more effective way to enforce cooperation than punishing rare cooperators is to enforce defection (since in the latter case the default behavior is to defect anyway, and so increasing the amount of “punishment” has little overall effect on payoff). And so as memory increases, cooperators become more robust relative to defectors, as 2–3 and Fig. 3 show.

The change in the efficacy of punishment for rare deviants from the social norm as memory capacity increases is illustrated in Fig. S2, where we calculate the average 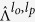 for randomly-drawn cooperative or defecting strategies. We see that as memory capacity increases, a randomly drawn cooperator tends to engage in more effective punishment (larger values of 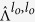) whereas a randomly drawn defector tends to engage in less effective punishment (smaller values of 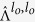). This trend explains why increasing memory capacity increases the volume of robust cooperators relative to defectors.

### Evolution of memory

Our results on the relationship between memory capacity and the robustness of cooperation raise a number of interesting questions. In particular, memory of the type we have considered does not seem to convey a direct advantage to cooperation (or defection), because a robust cooperative (or defecting) strategy is robust against *all possible invaders*, regardless of their memory capacity. However increased memory can nonetheless make robust cooperation easier to evolve, because it allows for more effective contingent punishment. This tends to have a stronger impact when games are small because, as described in Eqs 2, 3, the impact of contingent punishment on robustness is attenuated by a factor *N*–*n*, and thus the effect of longer memory on the contributions of 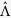 terms to robust cooperation is smaller in larger games. And so, at least when the number of players is relatively small, we might expect long memories to facilitate the evolution of cooperation in populations.

**Figure 4:**
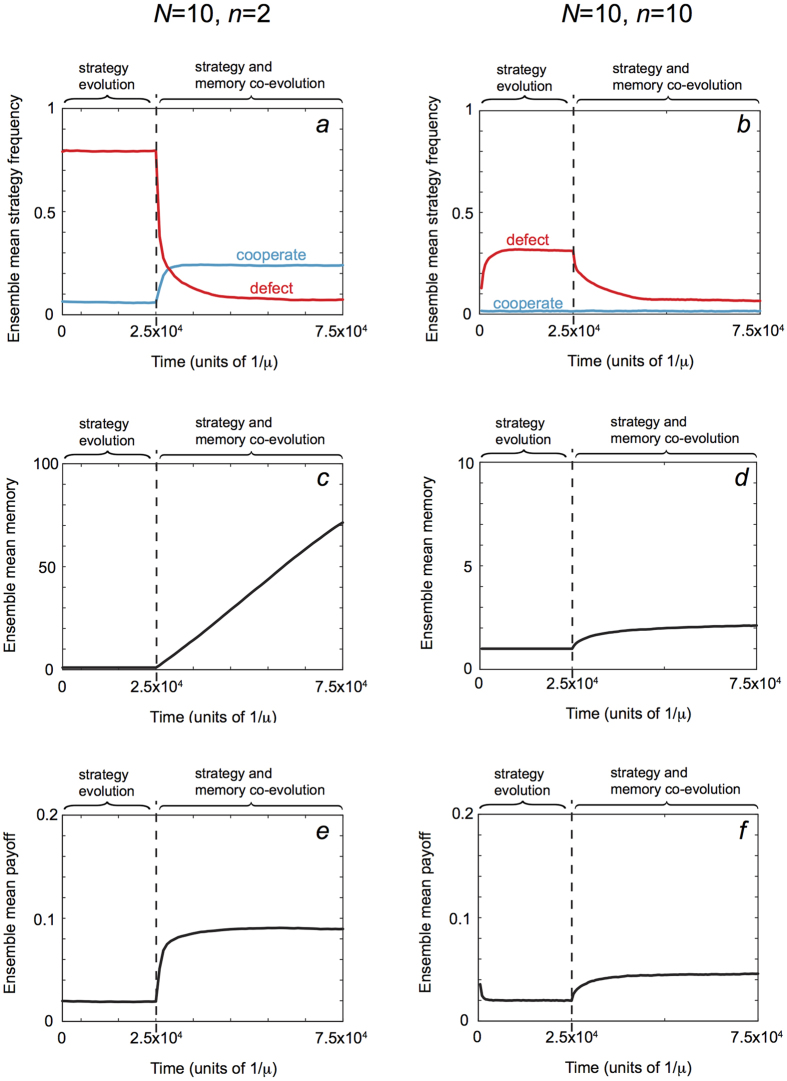
Co-evolution of strategies and memory capacity. We simulated populations playing the iterated *n*-player public-goods game, proposing mutant strategies until reaching equilibrium, and then also proposing mutations to a player’s memory capacity *m*, each at rate *μ*/10. In these simulations all players initially have memory *m* = 1, with payoff parameters *C* = 1 and *B* = 1.2. Mutations to strategies were drawn uniformly from the full space of memory-*m* strategies. Mutations perturbing the memory *m* caused it to increase or decrease by 1, with a lower bound of *m* = 1. Evolution was modeled according to a copying process under weak mutation^31^ in a population of size *N* = 10 individuals. (**a**) When the group size is small, *n* = 2, defecting strategies are initially dominant in the population, but they are quickly replaced by cooperators as memory capacity evolves to higher values. (**b**) When group size is large, *n *= *N *= 10, defecting strategies initially dominate the population and they remain dominant as memory evolves. In both (**a**,**b**) the overall frequency of cooperators and defectors (that is, the fraction of robust cooperative (and defecting) strategies among all cooperative and (defecting) strategies) decline as the dimension of strategy space increases, in line with the decline in the overall volume of robust strategies (Fig. S4). (**c**) When the game size is small memory evolves rapidly to larger values, reflecting the greater success of longer-memory strategies at invading (Fig. S3), and driving the increase in cooperative as compared to defecting strategies. (**d**) When the group size is large memory does not evolve to large values, reaching only *m* = 2 across 50,000 generations, and reflecting the decline in long-memory strategies’ success as invaders in larger games. (**e**) As cooperation increases so does the average payoff of the population, by a factor of 5–10 fold. (**f**) The lack of increase in cooperation results in a much more modest (although still appreciable) increase in average payoff for the population as defectors become less frequent.

What our analysis has not yet addressed is whether memory capacity itself can adapt, and what its co-evolution with strategies in a population will imply for the longterm prospects of cooperation. To address this question we undertook evolutionary simulations, allowing heritable mutations both to a player’s strategy and also to her memory capacity. These simulations, illustrated in Fig. 4, confirm that (i) longer memories do indeed evolve and (ii) this leads to an increase in the amount of cooperation in a population (Fig. 4). In a two-player game, memory tends to increase over time, which in turn drives an increase in the frequency of cooperators and a decline in defectors. This is accompanied by a large overall increase in the population mean fitness. By contrast, when the group size is large, *n* = *N*, there is little evolutionary change in memory capacity and defection continues to be more frequent than cooperation even as strategies and memory co-evolve. In a two-player game, even when memory comes at a substantial cost, an intermediate level of memory evolves, and there is a corresponding increase in the degree of cooperation (Fig. S3).

How are we to understand why memory evolves at all in these co-evolutionary simulations? The change in memory capacity is puzzling, at first glance, because a longer memory conveys no direct advantage against a resident robust strategy – since robustness implies uninvadability by any opponent, regardless of the opponent’s memory capacity. The key to understanding this co-evolutionary pattern is to note that longer memories are, on average, better at *invading* non-robust strategies, due to their greater capacity for contingent punishment (Fig. S3). Thus, when games are sufficiently small, the neutral drift that leads to turnover between cooperation and defection^31^,^34^ also provides opportunity for longer-memory strategies to invade and fix.

## Discussion

We have constructed a coordinate system that enables us to completely characterize the evolutionary robustness of arbitrary strategies in iterated multi-player public-goods games. This allows us to quantify the contrasting impacts of the number of players who engage in a game, and the memory capacity of those players on the evolution of cooperative behavior and collective action. In particular we have shown that while increasing the number of players in a game makes both cooperation and longer memories harder to evolve, in small groups, memory capacity tends to increase over time and drives the evolution of cooperative behavior.

To understand the evolution of social behavior it is not sufficient to simply determine whether particular types of strategies exist or not. Indeed, for repeated games, strategies that enforce any individually rational social norm are guaranteed to exist by the famous Folk Theorems^44^. The more incisive question, from an evolutionary viewpoint, is how often strategies of different types arise via random mutation, how often they reach fixation, and how long they remain fixed in the face of mutant invaders and other evolutionary forces such as neutral genetic drift. To address these questions we have analyzed the evolutionary robustness of strategies that result in sustained cooperation. We have shown that a strategy is more likely to be evolutionary robust if it can successfully punish defectors. We have shown that players with longer memories have access to a greater volume of such evolutionary robust strategies, and that, as a result, over the course of evolution populations that evolve longer memories are more likely to evolve cooperative behaviors. Memory of the type we have considered does not result in better strategies per se, but in a greater quantity of robust cooperative strategies.

In contrast to memory capacity, larger games favor defecting strategies over cooperating strategies, because larger games reduce the marginal cost to a player of switching from cooperation to defection, and make it harder for even long-memory players to effectively punish defectors. Thus we find in evolutionary simulations that only in small groups do both long-memory strategies and cooperation tend to evolve and dominate. The continued evolution of longer memories in small groups in our simulations is particularly striking, and reflects not the greater robustness of longer memory strategies, but their success as invaders.

It is important to emphasize that all of these effects of memory and group size are driven by changes in the volume of robust cooperative strategies. There is no single “best” strategy or memory length that evolution favors. Greater volumes of cooperative strategies lead to a greater degree of cooperation over the course of evolution in a population; and greater success as an invader among long-memory strategies leads to the evolution of longer memories. This is true *even when memory comes at a cost*, provided longer-memory strategies still enjoy increased success as invaders compared to shorter-memory strategies, which they often do (Fig. S3). According to the results of Press and Dyson^18^, a memory-1 player can always treat a longer-memory opponent as though he also has memory-1. And so when memory comes at a cost, it is always possible to construct a memory-1 strategy that outcompetes a longer memory strategy. This leads to opposing forces on the evolution of memory length, when memory comes at a cost, and complex evolutionary dynamics (Fig. S3).

How memory will evolve in natural populations depends on the genetic architecture of the organism and the magnitude of costs of memory^22^,^23^. Our results show that the evolution of cooperation in iterated games is strongly influenced by the memory capacity available to players, and therefore it cannot be adequately understood in general by restricting study to the space of memory-1 strategies, despite the results of Press and Dyson^18^.

A complex balance between behavior, memory, group size and environment can lead to wide variation in evolutionary outcomes in the presence of social interactions. Understanding this balance is vital if we are to understand and interpret the role of cooperative behavior in evolution. Despite the complexity of the problem, and the very general *n*-player memory-*m* setting we have analyzed, we have arrived at a few simple qualitative predictions, which may admit to testing not only in the social interactions of natural populations^12^ but also through experiments with human players^45^,^46^. Of course, the type of memory discussed here is only a small part of the story. We have ignored the possibility of other kinds of memory, which allow players to “tag” one another^26^,^27^ after the completion of a game. We have ignored the role of spatial structure, of demographic structure, and of dispersal^5^. We have failed to specify the underlying mechanisms by which public-goods and players’ decisions are produced and executed. Accounting for all of these additional factors is an important challenge as researchers seek to elucidate the emergence of collective action in evolving populations and beyond.

## Additional Information

**How to cite this article**: Stewart, A. J. and Plotkin, J. B. Small groups and long memories promote cooperation. *Sci. Rep.* **6**, 26889; doi: 10.1038/srep26889 (2016).

## Supplementary Material

Supplementary Information
