# Supplementary material for: Small groups and long memories promote cooperation
Source: Sci Rep. 2016 Jun 1;6:26889. doi: 10.1038/srep26889 (PMC4887980; doi:10.1038/srep26889)
Supplement: Supplementary Information [file srep26889-s1.pdf]

Small groups and long memories promote cooperation: Supporting information

Alexander J. Stewart*[1,2], Joshua B. Plotkin[1]

[1] Department of Biology, University of Pennsylvania, Philadelphia, PA 19104, USA

[2] Current address: Department of Genetics, Environment and Evolution, University College London, London, UK

* E-mail: alstew@sas.upenn.edu

1 Overview of Supporting Information

In this supplement we detail our analysis of iterated n-player games in which players have two choices in each round and can remember the outcomes of the previous m rounds. We identify the strategies that are able to resist selective invasion by any other strategy in an evolving population of players. Such strategies are called "evolutionary robust", as defined formally below. An iterated n-player game consists of an infinite series of "rounds" in each of which each player chooses to either "cooperate" (c) or "defect" (d). A memory-m strategy stipulates that the probability of cooperation in the current round depends on the outcomes of the preceding m rounds. The full space of memory-m strategies in such an n-player game thus has dimension $2^{n\times m}$. To identify strategies that are evolutionary robust across such a large space we first introduce a convenient coordinate transform for the space of memory-m strategies, which generalizes that introduced to study memory-1 strategies in iterated 2-player games [1–3]. This coordinate transformation enables us to identify sets of memory-m strategies that are robust to invasion by any other strategy in an evolving population. We apply this method to analyse evolutionary robustness in various n-player iterated public goods game.

1.1 Iterated n-player games

We consider an iterated game with an infinite number of successive rounds between a player, X_0 and her opponents $X_1, X_2 \ldots X_{n-1}$. We study games for which, in each round, each player has two choices, denoted cooperate (c) and defect (d). The payoffs in a given round to the focal player X_0 is given by

$R_{c,l-1}$, if she cooperates along with $l-1$ of her opponents, and it is given by $R_{d,l}$ if she defects while l of her opponents cooperate.

We will focus on public goods-type games, for which by definition in each round

- $R_{d,l} > R_{c,l-1}$ so that, given l players cooperating in total, those who defected receive a higher payoff than those who cooperated

- $R_{c,l} \geq R_{c,l-1}$ and $R_{d,l} \geq R_{d,l-1}$ so that, typically, the more of her opponents cooperate, the higher the payoff a cooperative focal player receives.

We will focus in particular on the most typical type of public goods game, for which $R_{c,l-1} = B\frac{l}{n} - C$ and $R_{d,l} = B\frac{l}{n}$, where $B > C$.

1.2 Memory-m strategies

A memory-m strategy takes account of the outcomes of the preceding m rounds of play among all players. As such in any given round there are $n \times m$ plays taken into account, and the strategy space therefore has dimension $2^{n\times m}$ – that is, a player's strategy consists of $2^{n\times m}$ probabilities for cooperation. First we develop notation to describe the probability that a focal player will cooperate in a focal round, given the plays made by all n players over the preceding m rounds. We denote the sequence of plays of the ith player over the preceding m rounds $\boldsymbol{\sigma}_i$, which has elements σ_k^i, denoting the play of player i, k steps in the past, where $i = 0 \ldots n-1$ and $k = 1 \ldots m$. Thus $\sigma_k^i = c$ if player i cooperated and $\sigma_k^i = d$ if she defected k steps in the past. We then write the probability for cooperation for a particular history of play in its most general form as $p_{\boldsymbol{\sigma}_0,\boldsymbol{\sigma}_1,\boldsymbol{\sigma}_2,\ ...\ ,\boldsymbol{\sigma}_{n-1}} \in [0,1]$.

In order to determine the robustness of such strategies, it will be convenient to introduce the operator θ which returns

$$\theta\left(\sigma_k^i\right) = \begin{cases} 1 & \text{if } \sigma_i^k = c \\ 0 & \text{if } \sigma_i^k = d \end{cases}$$

where for simplicity we will often write θ_k^i in place of $\theta\left(\sigma_k^i\right)$ for the play of the ith player k steps back in time. The number of times player i cooperated within memory is thus $\sum_{k=1}^{m} \theta_k^i$ and the number of players who cooperated in the immediately preceding round is $\sum_{i=0}^{n-1} \theta_1^i$.

1.3 Equilibrium payoffs in Iterated Games

The longterm scores received by n memory-m players in an infinitely iterated game are calculated from the equilibrium rates of the different plays. This can be determined from the stationary distribution a Markov chain on $2^{n\times m}$ states, which correspond to the history of plays across the preceding m rounds. In order to do this we write the equilibrium rate of a particular history of plays as $v_{\boldsymbol{\sigma}_0,\boldsymbol{\sigma}_1,\boldsymbol{\sigma}_2,\ ...\ ,\boldsymbol{\sigma}_{n-1}}$.

The essential trick we use to analyze equillibrium payoffs in multi-player games among players with long-memory strategies is to reduce the problem to an equivalent problem involving more players each using only memory-1 strategies. The advantage of the memory-1 setting is that it will allow us to express equillibrium payoffs in the framework of determinants developed by Press & Dyson and others **??**. In particular, given a game among n players who memory-m strategies we construct an equivalent $n \times m$-player game in which players use only memory-1 strategies. Of these $n \times m$ players, n are "real" players, and they each use a memory-1 strategy that corresponds precisely to a memory-m strategy in the original long-memory game, as described above. In order to allow the "real" memory-1 players to effectively react to the entire history of plays across m prior rounds we construct $m-1$ "shadow" players for each real player, who encode the information of earlier rounds. At each round, the shadow player with index $k > 1$ deterministically executes the play of its associated real player, k rounds in the past.

Given an n-player game among memory-m strategies, we encode the equivalent $n \times m$-player game among memory-1 strategies by writing $\mathbf{p}^{i,k}$ as the vector of all $2^{n\times m}$ probabilities for cooperation for the ith player, k steps in the past. If we order the players such that they are indexed from $j = 1 \ldots n \times m$ then the index of player i, k is given by $j = i \times m + k$. In this labelling system, $\mathbf{p}^{i,1}$ is the strategy of the ith real player, and $\mathbf{v}$ is the corresponding stationary vector of equilibrium rates of play. Finally we must encode the "strategy vector" of the shadow players, which encode how a player updates her memory each round. This is simple to do. We write $\mathbf{p}^{i,k}$ as the "strategy" vector which updates the memory of player i, k steps in the past (see Figure. S1 for illustration). This vector has entry 1 if $\theta^i_{k-1} = 1$ and 0 if $\theta^i_{k-1} = 0$. Thus the real strategy of player i consists of probabilities $\mathbf{p}^{i,1} \in [0,1]^{n\times m}$, whereas a shadow strategy, for which $k > 1$, consists of deterministic quantities $\mathbf{p}^{i,k} \in \{0,1\}^{n\times m}$.

The equilibrium score of player X_0 against players $X_1, X_2 ...X_{n-1}$ is calculated according to a particular form of determinant D defined below and written as:

$$S^0_1 = \frac{\mathbf{v}\cdot\mathbf{R}^0_1}{\mathbf{v}\cdot\mathbf{I}} = \frac{D\left(\mathbf{p}^{0,1},\mathbf{p}^{0,2}...\mathbf{p}^{0,m},\mathbf{p}^{1,1},\mathbf{p}^{1,2}...\mathbf{p}^{1,m}...\mathbf{p}^{n-1,1},\mathbf{p}^{n-1,2}...\mathbf{p}^{n-1,m},\mathbf{R}^{0,1}\right)}{D\left(\mathbf{p}^{0,1},\mathbf{p}^{0,2}...\mathbf{p}^{0,m},\mathbf{p}^{1,1},\mathbf{p}^{1,2}...\mathbf{p}^{1,m}...\mathbf{p}^{n-1,1},\mathbf{p}^{n-1,2}...\mathbf{p}^{n-1,m},\mathbf{I}\right)} \tag{1}$$

Note that in this expression have used the notation of the associated game with $n \times m$, memory-1 players, n of which correspond to the players in the original n-player, memory-m game. In this equation $\mathbf{I}$ denotes the identity vector of size $2^{n\times m}$, for which all elements are 1, and $\mathbf{R}^{0,1}$ denotes the payoff vector of player X_0. The payoff to player 0 in a given round depends only on her own play and the plays of the n other players in that round. In general, the payoffs received by player i, in the round that occurred k steps previously is determined from the payoff vector $\mathbf{R}^{i,k}$, which has $2^{n\times m}$ elements $R^{i,k}_{\boldsymbol{\sigma}_0,\boldsymbol{\sigma}_1,\boldsymbol{\sigma}_2,\ ...\ ,\boldsymbol{\sigma}_{n-1}}$ can be written as

$$R^{i,k}_{\boldsymbol{\sigma}_0,\boldsymbol{\sigma}_1,\boldsymbol{\sigma}_2,\ ...\ ,\boldsymbol{\sigma}_{n-1}} = \begin{cases} R_{c,\sum_{j=0}^{n-1}\theta^j_k} & \text{if } \theta^i_k = 1 \\ R_{d,\sum_{j=0}^{n-1}\theta^j_k} & \text{if } \theta^i_k = 0 \end{cases} \tag{2}$$

Where for the standard public goods game we can write $R_{c,\sum_{j=0}^{n-1}\theta^j_k} = \frac{b}{n}\sum_{j=0}^{n-1}\theta^j_k - c\theta^i_k$ and $R_{d,\sum_{j=0}^{n-1}\theta^j_k} = \frac{b}{n}\sum_{j=0}^{n-1}\theta^j_k$.

In general, the determinant $D\left(\mathbf{p}^{0,1},\mathbf{p}^{0,2}...\mathbf{p}^{0,m},\mathbf{p}^{1,1},\mathbf{p}^{1,2}...\mathbf{p}^{1,m}...\mathbf{p}^{n-1,1},\mathbf{p}^{n-1,2}...\mathbf{p}^{n-1,m},\mathbf{f}\right)$ arises from a generalization of the results of Press & Dyson, [1] for two-player games, and of [4, 5] for multi-player games, and gives the dot product between the stationary vector $\mathbf{v}$ and an arbitrary vector $\mathbf{f}$ which has elements $f_{\boldsymbol{\sigma}_0,\boldsymbol{\sigma}_1,\boldsymbol{\sigma}_2,\ ...\ ,\boldsymbol{\sigma}_{n-1}}$. In the example of a three player game with memory-1 strategies between players X_0, X_1 and X_2 with strategies $\mathbf{p}$, $\mathbf{q}$ and $\mathbf{r}$, the determinant is given by

$$D(\mathbf{p_x},\mathbf{q_y},\mathbf{r_z},\mathbf{f}) = \det\begin{bmatrix} -1+p_{c,c,c}q_{c,c,c}r_{c,c,c} & -1+p_{c,c,c}q_{c,c,c} & -1+p_{c,c,c}r_{c,c,c} & -1+p_{c,c,c} & -1+q_{c,c,c}r_{c,c,c} & -1+q_{c,c,c} & -1+r_{c,c,c} & f_{c,c,c} \\ p_{c,c,d}q_{c,c,d}r_{d,c,c} & -1+p_{c,c,d}q_{c,c,d} & p_{c,c,d}r_{d,c,c} & -1+p_{c,c,d} & q_{c,c,d}r_{d,c,c} & -1+q_{c,c,d} & r_{d,c,c} & f_{c,c,d} \\ p_{c,d,c}q_{d,c,c}r_{c,d,c} & p_{c,d,c}q_{d,c,c} & -1+p_{c,d,c}r_{c,d,c} & -1+p_{c,d,c} & q_{d,c,c}r_{c,d,c} & q_{d,c,c} & -1+r_{c,d,c} & f_{c,d,c} \\ p_{c,d,d}q_{d,c,d}r_{d,d,c} & p_{c,d,d}q_{d,c,d} & p_{c,d,d}r_{d,d,c} & -1+p_{c,d,d} & q_{d,c,d}r_{d,d,c} & q_{d,c,d} & r_{d,d,c} & f_{c,d,d} \\ p_{d,c,c}q_{c,d,c}r_{c,c,d} & p_{d,c,c}q_{c,d,c} & p_{d,c,c}r_{c,c,d} & p_{d,c,c} & -1+q_{c,d,c}r_{c,c,d} & -1+q_{c,d,c} & -1+r_{c,c,d} & f_{d,c,c} \\ p_{d,c,d}q_{c,d,d}r_{d,c,d} & p_{d,c,d}q_{c,d,d} & p_{d,c,d}r_{d,c,d} & p_{d,c,d} & q_{c,d,d}r_{d,c,d} & -1+q_{c,d,d} & r_{d,c,d} & f_{d,c,d} \\ p_{d,d,c}q_{d,d,c}r_{c,d,d} & p_{d,d,c}q_{d,d,c} & p_{d,d,c}r_{c,d,d} & p_{d,d,c} & q_{d,d,c}r_{c,d,d} & q_{d,d,c} & -1+r_{c,d,d} & f_{d,d,c} \\ p_{d,d,d}q_{d,d,d}r_{d,d,d} & p_{c,d,d}q_{c,d,d} & p_{c,d,d}r_{c,d,d} & p_{c,d,d} & q_{c,d,d}r_{c,d,d} & q_{c,d,d} & r_{c,d,d} & f_{d,d,d} \end{bmatrix}. \tag{3}$$

Eq. 1 can be used to calculate the scores received by n memory-1 players in a given game. However, there

are certain cases in which the Markov chain describing the iterated game has multiple absorbing states, and the denominator of Eq.1 goes to zero. The scores in these cases can be calculated by assuming that players execute their strategy with some small "error rate" ϵ [6], so that the probability of cooperation is at most $1-\epsilon$ and at least ϵ. Assuming this, and taking the limit $\epsilon \to 0$ then gives the player's scores in the cases where multiple absorbing states exist.

2 Evolution in a population of players

We study the evolution of memory-m strategies in a population of N individuals playing an iterated n-player game, with $N \geq n$. In each generation, all subsets of n players in the population engage in the iterated game, and each player in the population receives a total score across all the $\binom{N-1}{n-1}$ games in which she participates. We assume that the population is well-mixed, so that the makeup of different strategies these games depends upon the frequencies of strategies in the population. We focus on evolution under weak-mutation, in which a strategy X is resident in the population; a single mutant strategy Y arises through mutation; and Y is subsequently either lost or goes to fixation in the population, before another mutant arises. We always use X to denote the resident, and Y the mutant, strategy. Under this weak-mutation assumption there are at most two strategy types present in the population at any time.

We use the notation S_a^X to denote the payoff to strategy X in a single iterated game involving a players of type Y and $n-a$ players of type X. We use the notation S_a^Y to denote the payoff to strategy Y in a single iterated game involving a players of type Y and $n-a$ players of type X. When the population as a whole contains b players of type Y and $N-b$ players of type X, then, the total score to a player of type X, denoted $T^X(b)$, is given by

$$T^X(b) = \frac{(N-n)!(n-1)!}{(N-1)!} \sum_{a=0}^{\min[b,n-1]} \frac{(N-1-b)!}{(N-b-(n-a))!(n-1-a)!} \frac{b!}{a!(b-a)!} S_a^X$$

where the sum over a denotes the different number of opponents of type Y that X may face in the n-player games she plays in a single generation. The total score to a mutant Y in such a population, denoted $T^Y(b)$, can be calculated in the same way.

We model evolution according to the copying process [7], in which pairs of players are drawn at random from the population, and the first player switches her strategy to that of the second player with a probability that depends on the difference between their total scores. Thus a player using a strategy X

switches to Y with probability

$$f_{X \to Y} = \frac{1}{1 + \exp\left[s\left(T^X(a) - T^Y(a)\right)\right]}$$

where s is a parameter denoting the strength of selection.

The "strong-selection" regime of this process occurs when $Ns \gg 1$. Under this regime selection is sufficiently strong that an invading mutant is extremely unlikely to reach high frequency in the populaiton, unless it has a selecive advantage (or is neutral) against the resident strategy in the population. Thus under strong selection, resident strategies that can resist invasion by all other mutants are evolutionary robust. Alternatively, the "weak-selection" limit arises when $Ns \ll 1$ in which case even deleterious strategies may reach high frequency through genetic drift. We focus on the regime of strong selection in our analysis below.

2.1 Evolutionary robustness

The concept of evolutionary robustness [8] is similar to the notion of evolutionary stability [9, 10], but more useful for studying evolution in large strategy spaces, in which an ESS strategy typically does not exist [3, 8]. In general, a strategy is defined to be evolutionary robust if, when resident in a population, there is no mutant that is favored to spread by natural selection when rare [8].

More precisely, under strong selection a resident strategy X is evolutionary robust iff $T^Y(1) \leq T^X(1)$ for all strategies Y. This condition for evolutionary robustness under strong selection is identical to that of a Nash equilibrium in the limit $N \to \infty$.

3 Coordinate Transform

In two-player games, the work of Press & Dyson [1] and Akin [2] allows us to identify a coordinate transform for the full space of memory-1 strategies. This coordinate transformation permits a simple closed-form expression relating the scores of two players in a game, which has enabled us to identify all evolutionary robust memory-1 strategies, under both strong and weak selection [3,8]. Here we extend this line of analysis to multi-player games with memory-m strategies. We begin by identifying an analogous coordinate transform for the $2^{n\times m}$-dimensional space of memory-m strategies in an n-player game.

To define the desired co-ordinate transform we must identify $2^{n\times m}$ vectors that form a basis in $\mathbb{R}^{n\times m}$

and that allow us to write down a simple, closed-form relationship between the players' scores in a given game.

These vectors consist firstly of the $n \times m + 1$ vectors $\mathbf{R}_k^i$ for each player's payoff in the kth preceding round, along with the identify vector $\mathbf{I}$, with entry 1 in all positions. The second set of vectors in the coordinate transform consists of the $n \times m - 1$ vectors denoted $\mathbf{L}^l$, where $\mathbf{L}^l$ is has entry 1 when l players cooperated in the previous m rounds and entry 0 otherwise, regardless of the focal player's play in the previous round. Note that this excludes the case where all players cooperated and the case where no players cooperated, which are accounted for by the identity vector.

The final $2^{n \times m} - 2n \times m$ vectors required for the coordinate transform account for the degeneracy that arises due to the number of ways in which l players can cooperate in the preceding m rounds. Given l, if the focal player cooperates l_p times over m rounds, and her opponents therefore cooperate $l_o = l - l_p$ times, she will receive the same total payoff over those m rounds in a standard public goods game, regardless of which players cooperated or when. In the most general case a player may nonetheless distinguish between the play of each player (including herself) in each of the preceding m rounds.

We already have $2n \times m$ vectors, as described above. The simplest way to account for the remaining dimensions (required for players to distinguish between all possible outcomes) is simply to add

$$2 \sum_{l=1}^{n \times m - 1} \left(\frac{(n \times m - 1)!}{k!(n \times m - l - 1)!} - 1 \right) = 2^{n \times m} - 2n \times m$$

vectors, denoted $\mathbf{G}^{l_o, l_p}_{\boldsymbol{\sigma}_0, \boldsymbol{\sigma}_1, \boldsymbol{\sigma}_2, \ldots, \boldsymbol{\sigma}_{n-1}}$, which have entry 1 for a single set of plays $(\boldsymbol{\sigma}_0, \boldsymbol{\sigma}_1, \boldsymbol{\sigma}_2, \ldots, \boldsymbol{\sigma}_{n-1})$, for which $\sum_{i=1}^{n-1} \sum_{k=1}^{m} \theta_k^i = l_o$ is the total number of times all players have cooperated in the last m rounds and $\sum_{k=1}^{m} \theta_k^0 = l_p$ is the number of times the focal player has cooperated in the last m rounds. Note that we have written these vectors in terms of l_p and l_o in order to aid later analysis.

We adopt the convention that we do not add a vector $\mathbf{G}^{l_o, l_p}_{\boldsymbol{\sigma}_0, \boldsymbol{\sigma}_1, \boldsymbol{\sigma}_2, \ldots, \boldsymbol{\sigma}_{n-1}}$ for the set of opponent plays which is ordered *cccc. . .dddd* across the ordered history of all players, and the set focal player plays ordered either *cccc. . .dddd* or *dccc. . .dddd*. That is, the play for which the first l_o terms of the sum $\sum_{i=1}^{n-1} \sum_{k=1}^{m} \theta_k^i$ are 1, and either $\theta_0^1 = 1$ and $\sum_{k=1}^{l_p} \theta_k^i = l_p$ or else $\theta_0^1 = 0$ and $\sum_{k=1}^{l_p+1} \theta_k^i = l_p$.

In summary, the $2^{n \times m}$ vectors for the coordinate transform consist of

- $n \times m$ vectors $\mathbf{R}$ for the player's payoffs, and the vector $\mathbf{I}$.

- $n \times m - 1$ vectors $\mathbf{L}^l$ with entry 1 when l players cooperated in the previous round.
- $2^{n \times m} - 2n \times m$ vectors $\mathbf{G}$ with a single entry 1, to account for the degeneracy which arises when different combinations of opponents cooperate.

Although this is a somewhat complex transformation, we shall see the utility of working this way in what follows.

For clarity's sake we can write down this coordinate system explicitly, first for the case of $n = 3$ players with memory-1. The new coordinate system is $\left\{\mathbf{R}_1^0, \mathbf{R}_1^1, \mathbf{R}_1^2, \mathbf{I}, \mathbf{L}^1, \mathbf{L}^2, \mathbf{G}_{c,d,c}^{1,1}, \mathbf{G}_{d,d,c}^{1,0}\right\}$ and we have

$$\det \begin{bmatrix} R_{c,2} & R_{c,2} & R_{c,2} & 1 & 0 & 0 & 0 & 0 \\ R_{c,1} & R_{c,1} & R_{d,2} & 1 & 1 & 0 & 0 & 0 \\ R_{c,1} & R_{d,2} & R_{c,1} & 1 & 1 & 0 & 1 & 0 \\ R_{c,0} & R_{d,1} & R_{d,1} & 1 & 0 & 1 & 0 & 0 \\ R_{d,2} & R_{c,1} & R_{c,1} & 1 & 1 & 0 & 0 & 0 \\ R_{d,1} & R_{c,0} & R_{d,1} & 1 & 0 & 1 & 0 & 0 \\ R_{d,1} & R_{d,1} & R_{c,0} & 1 & 0 & 1 & 0 & 1 \\ R_{d,0} & R_{d,0} & R_{d,0} & 1 & 0 & 0 & 0 & 0 \end{bmatrix} = -3(R_{c,2} - R_{d,0})(R_{d,1} - R_{c,0})(R_{d,2} - R_{c,1}) \tag{4}$$

which is therefore a basis $\mathbb{R}^8$. Similarly, for the case of $n = 2$ players with with memory-m the new coordinate system is $\left\{\mathbf{R}_1^0, \mathbf{R}_1^1, \mathbf{R}_2^0, \mathbf{R}_2^1, \mathbf{I}, \mathbf{L}^1, \mathbf{L}^2, \mathbf{L}^3, \mathbf{G}_{dd,cd}^{1,0}, \mathbf{G}_{dd,dc}^{1,0}, \mathbf{G}_{dd,cc}^{2,0}, \mathbf{G}_{dc,dc}^{1,1}, \mathbf{G}_{cd,cd}^{1,1}, \mathbf{G}_{cd,dc}^{1,1}, \mathbf{G}_{cc,dc}^{1,2}, \mathbf{G}_{cd,cc}^{2,1}\right\}$ and we have

$$\det\begin{bmatrix}
R_{c,2} & R_{c,2} & R_{c,2} & R_{c,2} & 1 & 0 & 0 & 0 & 0 & 0 & 0 & 0 & 0 & 0 & 0 & 0\\
R_{c,2} & R_{c,1} & R_{c,2} & R_{d,1} & 1 & 0 & 0 & 1 & 0 & 0 & 0 & 0 & 0 & 0 & 0 & 0\\
R_{c,2} & R_{d,1} & R_{c,2} & R_{c,1} & 1 & 0 & 0 & 1 & 0 & 0 & 0 & 0 & 0 & 0 & 0 & 1\\
R_{c,2} & R_{d,0} & R_{c,2} & R_{d,0} & 1 & 0 & 1 & 0 & 0 & 0 & 0 & 0 & 1 & 0 & 0 & 0\\
R_{c,1} & R_{c,2} & R_{d,1} & R_{c,2} & 1 & 0 & 0 & 1 & 0 & 0 & 0 & 0 & 0 & 0 & 1 & 0\\
R_{c,1} & R_{c,1} & R_{d,1} & R_{d,1} & 1 & 0 & 1 & 0 & 0 & 0 & 0 & 0 & 0 & 0 & 0 & 0\\
R_{c,1} & R_{d,1} & R_{d,1} & R_{c,1} & 1 & 0 & 1 & 0 & 0 & 0 & 0 & 0 & 0 & 1 & 0 & 0\\
R_{c,1} & R_{d,0} & R_{d,1} & R_{d,0} & 1 & 1 & 0 & 0 & 0 & 0 & 0 & 0 & 0 & 0 & 0 & 0\\
R_{d,1} & R_{c,2} & R_{c,1} & R_{c,2} & 1 & 0 & 0 & 1 & 0 & 0 & 0 & 0 & 0 & 0 & 0 & 0\\
R_{d,1} & R_{c,1} & R_{c,1} & R_{d,1} & 1 & 0 & 1 & 0 & 0 & 0 & 0 & 0 & 0 & 0 & 0 & 0\\
R_{d,1} & R_{d,1} & R_{c,1} & R_{c,1} & 1 & 0 & 1 & 0 & 0 & 0 & 1 & 0 & 0 & 0 & 0 & 0\\
R_{d,1} & R_{d,0} & R_{c,1} & R_{d,0} & 1 & 1 & 0 & 0 & 1 & 0 & 0 & 0 & 0 & 0 & 0 & 0\\
R_{d,0} & R_{c,2} & R_{d,0} & R_{c,2} & 1 & 0 & 1 & 0 & 0 & 0 & 0 & 1 & 0 & 0 & 0 & 0\\
R_{d,0} & R_{c,1} & R_{d,0} & R_{d,1} & 1 & 1 & 0 & 0 & 0 & 0 & 0 & 0 & 0 & 0 & 0 & 0\\
R_{d,0} & R_{d,1} & R_{d,0} & R_{c,1} & 1 & 1 & 0 & 0 & 0 & 1 & 0 & 0 & 0 & 0 & 0 & 0\\
R_{d,0} & R_{d,0} & R_{d,0} & R_{d,0} & 1 & 0 & 0 & 0 & 0 & 0 & 0 & 0 & 0 & 0 & 0 & 0
\end{bmatrix} = -4(R_{c,2}-R_{d,0})^2(R_{d,1}-R_{c,1})^2 \tag{5}$$

which is therefore a basis $\mathbb{R}^{16}$.

Using the results of Press & Dyson, generalised to multi-player games [1,4,5], the strategy of the focal player in this coordinate new system, which for convenience we assign index $i = 0$, is given by a vector of the form:

$$\mathbf{p}^{0,1} - \boldsymbol{\theta}_1^0 = \sum_{i=0}^{n-1}\sum_{k=1}^{m} \alpha_k^i \mathbf{R}_k^i + \alpha_n \mathbf{1} + \sum_{l_o=0}^{(n-1)\times m}\sum_{l_p=0}^{m}\left[\lambda_{l_o+l_p}\mathbf{L}^{l_o+l_p} + \sum_{\boldsymbol{\sigma}\in\mathcal{H}_{o,p}} \gamma_{\boldsymbol{\sigma}}^{l_o,l_p}\mathbf{G}_{\boldsymbol{\sigma}}^{l_o,l_p}\right]. \tag{6}$$

where we define $\mathcal{H}_{o,p} = \left\{(\sigma_0,\ldots,\sigma_n) | \sum_{k=1}^{m}\theta_k^0 = l_p, \sum_{i=1}^{n-1}\sum_{k=1}^{n-1}\theta_k^i = l_o\right\}$ is the set of combinations of plays by n players across the last m rounds such that player 0 (the focal player) cooperated l_p times and her oppoennts cooperated a total l_o times. Note that we set $\lambda = \gamma = 0$ when $l_o = l_p = 0$ and $l_o + l_p = n \times m$. The vector $\boldsymbol{\theta}_1^0$ has the corresponding elements θ_1^0 for the play of the focal player in the

preceding round (i.e 1 if she cooperated and 0 if she defected), and we have written $\sigma = (\sigma_0, \ldots, \sigma_n)$.

From Eq.1 the scores of the players are then related by the expression

$$\sum_{i=0}^{n-1}\sum_{k=1}^{m} \alpha_k^i S^i + \alpha_n \mathbf{1} + \sum_{l_o=0}^{(n-1)\times m}\sum_{l_p=0}^{m}\left[\lambda_{l_o+l_p} v^{l_o,l_p} + \sum_{\boldsymbol{\sigma}\in\mathcal{H}_{o,p}} \gamma_{\boldsymbol{\sigma}}^{l_o,l_p} v_{\boldsymbol{\sigma}}^{l_o,l_p}\right] = 0. \qquad (7)$$

where S^i denotes the equilibrium score of player i in the current game, $v^{l_o+l_p}$ denotes the rate at which $l_o + l_p$ players cooperate and $v_{\boldsymbol{\sigma}}^{l_o,l_p}$ is the rate at which the focal player cooperates l_p times, along with l_o of her opponents, with the sequence of plays following the ordering $\boldsymbol{\sigma} = (\boldsymbol{\sigma}_0, \boldsymbol{\sigma}_1, \boldsymbol{\sigma}_2, \ldots, \boldsymbol{\sigma}_{n-1})$. Note that the equilibrium score of player i is independent of k in Eq. 7.

We now additionally define the parameters $\chi_k^0 = -\alpha_k^0$; $\phi_{i\times m+k} = \alpha_k^i$; and $\kappa\left(\sum_{k=1}^{m}\left(\chi_k^0 - \sum_{i=1}^{n}\phi_{i\times m+k}\right)\right) = \alpha_n$. In this new parameterization we can re-write the relationship among the players' scores as:

$$\sum_{k=1}^{m}\left(\sum_{i=1}^{n-1}\phi_{i\times m+k}(S^i-\kappa) - \chi_k^0(S^0-\kappa)\right) + \sum_{l_o=0}^{(n-1)\times m}\sum_{l_p=0}^{m}\left[\lambda_{l_o+l_p} v^{l_o,l_p} + \sum_{\boldsymbol{\sigma}\in\mathcal{H}_{o,p}} \gamma_{\boldsymbol{\sigma}}^{l_o,l_p} v_{\boldsymbol{\sigma}}^{l_o,l_p}\right] = 0 \qquad (8)$$

Eq.8 gives the most general form for the relationship between player's scores in an n-player game with memory-m. Henceforth we will restrict our analysis restricted to a focal strategy in which a memory-m player does not distinguish between her opponents, and does not pay attention to the order of cooperation events. As such we consider a focal player who keeps track of two quantities: (i) the total number of times her opponents cooperated in the last m rounds and (ii) the total number of times she cooperated in the last m rounds.

3.1 Strategies that track cooperation frequency

If a focal player tracks only the number of times she cooperated in the last m rounds, and the total number of times her opponent cooperated in the last m rounds, then her memory-m strategy consists of $((n-1)m+1) \times (m+1)$, since her $(n-1)$ opponents can cooperate anywhere between 0 and $(n-1)m$ times in m rounds, and she can cooperate anywhere between 0 and m times, to give a strategy consisting of $((n-1)m+1) \times (m+1)$ probabilities for cooperation. We will henceforth explicitly adopt a standard

public goods payoff structure, with $R_{c,l} = B\frac{l}{n} - C$ and $R_{d,l} = B\frac{l}{n}$

Eq. 6 encodes a strategy with $2^{n\times m}$ probabilities for cooperation, many of which are redundant in our reduced strategy space. Let the focal player cooperate l_p times and her opponents cooperate l_o times in m rounds.

Starting from Eq. 8 are now able to make two observations:

(i) If the focal player does not distinguish between opponents, or the order in which they cooperate, then her payoff, and the equilibrium rate of play v^{l_o,l_p} are the same for all $((n-1)\times m)! \times m!$ orderings of opponents and plays. Summing over all possible orderings of opponents and dividing by $((n-1)\times m)!\times m!$ results in the equilibrium scores being related by

$$\phi\sum_{j=1}^{n-1}\frac{S^j}{n-1} - \sum_{k=1}^{m}\chi_k^0(S^0-\kappa) - \kappa\phi + \sum_{l_o=0}^{(n-1)\times m}\sum_{l_p=0}^{m}\left[\lambda_{l_o+l_p} + \gamma^{l_o,l_p}\right]v^{l_o,l_p} = 0 \tag{9}$$

where we have set $\phi = \sum_{i=1}^{n-1}\sum_{k=1}^{m}\phi_{i\times m+k}$ and $\frac{l_p!(m-l_p)!}{m!}\frac{l_o!((n-1)\times m-l_o)!}{((n-1)\times m)!}\sum_{\boldsymbol{\sigma}\in\mathcal{H}_{o,p}}\gamma_{\boldsymbol{\sigma}}^{l_o,l_p} = \gamma^{l_o,l_p}$, and the γ terms result from noting that, when summing over all orderings of opponents and events, a given term γ^{l_o,l_p} is multiplied by each rate of play $v_{\boldsymbol{\sigma}}^{l_o,l_p}$ a total

$$\frac{m!}{l_p!(m-l_p)!}\frac{((n-1)\times m)!}{l_o!((n-1)\times m-l_o)!}$$

(ii) If we then sum over all $\frac{m!}{l_p!(m-l_p)!}\frac{((n-1)\times m)!}{l_o!((n-1)\times m-l_o)!}$ degenerate probabilities for a given l_o and l_p we then arrive at

$$-\frac{l_p}{m} + p^{l_o,l_p} = -\frac{l_o}{(n-1)\times m}C\phi + B\frac{l_o+l_p}{n\times m}\left(\phi - \sum_{k=1}^{m}\chi_k^0\right) + \sum_{k=1}^{m}\chi_k^0 C\frac{l_p}{m} - \left(\phi - \sum_{k=1}^{m}\chi_k^0\right)\kappa + \lambda_{l_o+l_p} + \gamma^{l_o,l_p}.$$

as the expression for the probability of cooperation given that the focal player cooperated l_p times and her opponents l_o times in the last m rounds, assuming she only tracks cooperation frequency.

3.2 Boundary conditions

If we recall our convention that the equation lacking a γ is that which is ordered with *cccc.....dddd* etc we then have the following boundary conditions

$$-1+p^{l_o,m} = -C\sum_{i=1}^{l_o}\phi_i + B\frac{l_o+l_p}{n\times m}\left(\sum_{i=1}^{l_o}\phi_i - \sum_{k=1}^{m}\chi_k^0\right) + \sum_{k=1}^{m}\chi_k^0 C - \left(\sum_{i=1}^{l_o}\phi_i - \sum_{k=1}^{m}\chi_k^0\right)\kappa + \lambda_{l_o+m}$$

and

$$-1+p^{0,l_p} = B\frac{l_p}{n\times m}\left(\sum_{i=1}^{l_o}\phi_i - \sum_{k=1}^{m}\chi_k^0\right) + \sum_{k=1}^{l_p}\chi_k^0 C - \left(\sum_{i=1}^{l_o}\phi_i - \sum_{k=1}^{m}\chi_k^0\right)\kappa + \lambda_{l_p}$$

Similarly the term with *dccc.....dddd* lacks a γ terms so that

$$p^{l_o,m-1} = -C\sum_{i=1}^{l_o}\phi_i + B\frac{l_o+l_p}{n\times m}\left(\sum_{i=1}^{l_o}\phi_i - \sum_{k=1}^{m}\chi_k^0\right) + \sum_{k=2}^{m}\chi_k^0 C - \left(\sum_{i=1}^{l_o}\phi_i - \sum_{k=1}^{m}\chi_k^0\right)\kappa + \lambda_{l_o+m-1}$$

and

$$p^{0,l_p} = B\frac{l_p}{n\times m}\left(\sum_{i=1}^{l_o}\phi_i - \sum_{k=1}^{m}\chi_k^0\right)\phi + \sum_{k=2}^{l_p+1}\chi_k^0 C - \left(\sum_{i=1}^{l_o}\phi_i - \sum_{k=1}^{m}\chi_k^0\right)\kappa + \lambda_{l_p}$$

First, combining the two expressions for p^{0,l_p} gives

$$\phi C\chi_{l_p+1}^0 = 1 + \phi C\chi_1^0$$

and since this must hold for all l_p we have $\chi_{l_p+1}^0 = \chi_m^0$ is constant, and

$$\phi = \frac{1}{C(\chi_m^0 - \chi_1^0)} \tag{10}$$

Substituting these into the general expression for p^{0,l_p} we find

$$\gamma^{0,l_p} = 0$$

Second, combining the expressions for $p^{l_o,m}$ and $p^{l_o,m-1}$ gives

$$\phi\gamma^{l_o,m} = \frac{m-1}{m} + \phi C \frac{m-1}{m}(\chi_1^0 - \chi_m^0) + \phi\gamma^{l_o,m-1}$$

Substituting for ϕ we then find

$$\gamma^{l_o,m} = \gamma^{l_o,m-1}$$

Finally, since $\gamma^{(n-1)\times,m} = 0$ by definition this also implies

$$\gamma^{(n-1)\times m,m-1} = 0$$

We therefore have $((n-1)\times m+1)(m+1)-(n\times m+2)$ parameters γ^{l_o,l_p}, plus $n\times m-1$ parameter λ_{l_o,l_p}, plus 3 parameters χ, ϕ and κ to give a total $((n-1)\times m+1)(m+1)$ parameters as required. We can use these boundary conditions for γ to construct the inverse coordinate transform. We arrive at the three simultaneous equations which can be solved for for κ, χ and ϕ:

$$\sum_{i=1}^{(n-1)\times m-1}\left(p^{i,m} - p^{i,m-1}\right) - (p^{(n-1)\times m,m-1} - p^{0,m}) = (n-1) - C(n-1)\chi - C\phi$$
$$p^{0,0} = \kappa(\phi - \chi)$$
$$p^{(n-1)\times m,m} = 1 + \kappa(\phi-\chi) - (B-C)(\phi-\chi)$$

with the remaining terms Λ^{l_o,l_p} being determined by these three parameters plus p^{l_o,l_p}. Finally, we set

$$\chi = \chi_1^0 + (m-1)\chi_m^0 \tag{11}$$

and

$$\Lambda^{l_o,l_p} = \lambda_{l_o+l_p} + \gamma^{l_o,l_p} \tag{12}$$

to define a coordinate system characterized by a vector of $((n-1)\times m+1)(m+1)$ numbers, $(\kappa, \chi, \phi, \Lambda^{0,0}, \ldots, \Lambda^{(n-1)\times m, m})$ where we have conditions $\Lambda^{0,0} = \Lambda(n-1)\times m, m = 0$ and a third linear condition as described above.

3.3 Strategies and payoffs in a public goods game

We can now write the relationship between the players' scores when players do not pay attention to the identity of their opponents as

$$\phi \sum_{i=1}^{n-1} \frac{S^i}{n-1} - \chi(S^0 - \kappa) - \phi\kappa + \sum_{l_o=0}^{(n-1)\times m} \sum_{l_p=0}^{m} \Lambda^{l_o,l_p} v^{l_o,l_p} = 0.$$

In the case when one player uses strategy Y and the rest use strategy X we then have the following relationship between scores:

$$\phi S^Y \frac{1}{n-1} + \phi S^X \frac{n-2}{n-1} - \chi(S^X - \kappa) - \phi\kappa + \sum_{l_o=0}^{(n-1)\times m} \sum_{l_p=0}^{m} \Lambda^{l_o,l_p} v^{l_o,l_p} = 0. \tag{13}$$

The strategy of a focal player in a public goods game can then be written as

$$p^{l_o,l_p} = \frac{l_p}{m} + \kappa(\phi - \chi) + \left(B\frac{l_o + l_p}{n\times m} - C\frac{l_p}{m}\right)\chi - \left(B\frac{l_o + l_p}{n\times m} - C\frac{l_o}{(n-1)\times m}\right)\phi - \Lambda^{l_o,l_p} \tag{14}$$

Since a viable strategy must have $0 \leq p^{l_o,l_p} \leq 1$ we see by looking at $p^{0,0}$ and $p^{(n-1)\times m,m}$ that

$$0 \leq \kappa \leq B - C$$

and

$$\phi > \chi$$

with additional constraints on the other parameters. This in turn implies that a cooperator, for which $p^{(n-1)\times m,m} = 1$ necessitates $\kappa = B - C$ and a defector, for which $p^{0,0} = 0$ necessitates $\kappa = 0$.

4 Equilibrium rates of play

We now derive some inequalities that, in combination with Eq. 11, will allow us to identify the strategies that are evolutionary robust in n-player games. In general, we can write the score of a focal player with resident strategy X as

$$S^X = \sum_{l_o=0}^{(n-1)\times m} \sum_{l_p=0}^{m} \left(B \frac{l_o + l_p}{n \times m} - C \frac{l_p}{m} \right) v^{l_o,l_p}$$

Similarly the score of an opponent with a strategy Y, is given by

$$S^Y = \sum_{l_o=0}^{(n-1)\times m} \sum_{l_p=0}^{m} \left(B \frac{l_o + l_p}{n \times m} - C \frac{l_p}{m} \right) w^{l_o,l_p}$$

where w^{l_o,l_p} are the equilibrium rates of play from Y's perspective. When there is only a single Y mutant in a game, then from Y's perspective, all opponents are identical and use strategy X. In this situation we can write

$$v^{l_o,l_p} = \sum_{l'_p=\max[0,l_o+l_p-(n-1)\times m]}^{\min[m,l_o]} w^{l_o+l_p-l'_p,l'_p} \frac{((n-1)\times m - l_o - l_p + l'_p)!}{(m-l_p)!((n-2)\times m - l_o + l'_p)!} \frac{(l_o+l_p-l'_p)!}{l_p!(l_o-l'_p)!} \frac{m!((n-2)\times m)!}{((n-1)\times m)!} \tag{15}$$

where we assume $w^{l_o+l_p-l'_p,l'_p} = 0$ for the unphysical case $l'_p > l_o$. This allows us to write the score of X in terms of w^{l_o,l_p}. We will now use these results to explore two special cases of interest: (i) The effect of increasing the size of the game n with fixed memory, and (ii) the effect of increasing memory size m with fixed game size.

5 Bounds on players' scores

We can now use Eq. 14 to find upper and lower bounds on the difference and the sum of players' scores, in the case that the game contains a single player using a strategy Y and $n-1$ players using a strategy X.

$$S^X = \sum_{l_o=0}^{(n-1)\times m} \sum_{l_p=0}^{m} \left(B\frac{l_o+l_p}{n\times m} - C\frac{l_o}{(n-1)\times m} \right) w^{l_o,l_p}$$

We can now write the difference between the scores of X and Y scores as

$$S^X - S^Y = \sum_{l_o=0}^{(n-1)\times m} \sum_{l_p=0}^{m} C\frac{l_p(n-1)-l_o}{(n-1)\times m} w^{l_o,l_p}$$

which enables us to identify upper and lower bounds on the difference between two players' scores, namely

$$S^X - S^Y \geq -\sum_{l_o=0}^{(n-1)\times m} \sum_{l_p=0}^{m} C\frac{l_o}{(n-1)\times m} w^{l_o,l_p} \tag{16}$$

which becomes an equality when Y always defects at equilibrium and

$$S^X - S^Y \leq \sum_{l_o=0}^{(n-1)\times m} \sum_{l_p=0}^{m} C\frac{(n-1)\times m - l_o}{(n-1)\times m} w^{l_o,l_p} \tag{17}$$

which becomes an equality when Y never defects at equilibrium. We can similarly write, for the sum of the player's scores,

$$S^X + S^Y \frac{1}{n-1} = \sum_{l_o=0}^{(n-1)\times m} \sum_{l_p=0}^{m} (B-C) \frac{l_o + l_p}{(n-1)\times m} w^{l_o,l_p}.$$

This gives an upper bound on the sum

$$S^X + S^Y \frac{1}{n-1} \leq \frac{n}{n-1}(B-C) - \sum_{l_o=0}^{(n-1)\times m} \sum_{l_p=0}^{m} (B-C) \frac{n\times m - l_o - l_p}{(n-1)\times m} w^{l_o,l_p}. \tag{18}$$

which becomes an equality when $w^{0,0} = 0$ at equilibrium (i.e it is never the case that all players defect). Finally we have a lower bound on the sum

$$S_1^X + S_1^Y \frac{1}{n-1} \geq \sum_{l_o=0}^{(n-1)\times m} \sum_{l_p=0}^{m} (B-C) \frac{l_o + l_p}{(n-1)\times m} w^{l_o,l_p}. \tag{19}$$

which becomes an equality when $w^{(n-1)\times m,m} = 0$, (i.e it is never the case that all players cooperate at equilibrium).

It is also convenient to rewrite Eq. 12 for the relationship between two player's scores in terms of w to give

$$\phi S^Y \frac{1}{n-1} + \phi S^X \frac{n-2}{n-1} - \chi(S^X - \kappa) - \phi\kappa +$$
$$\sum_{l_o=0}^{(n-1)\times m} \sum_{l_p=0}^{m} \left(\sum_{k=\max[0,l_o+l_p-(n-1)\times m]}^{\min[m,l_o]} \Lambda^{l_o+l_p-k,k} \frac{((n-1)\times m - l_o)!}{(m-k)!((n-2)\times m - l_o + k)!} \frac{l_o!}{k!(l_o-k)!} \frac{m!((n-2)\times m)!}{((n-1)\times m)!} \right) w^{l_o,l_p} = 0. \tag{20}$$

We can now use Eqs. 16-20 to identify the strategies that are evolutionary robust, under strong selection, in multi-player games.

5.1 Robust strategies

We focus here on the prospects for cooperation in iterated games. In particular, we identify strategies which, when used by all players in a game, ensure that all players cooperate. This is achieved quite simply by setting $p^{m\times(n-1),m} = 1$ so that if all players cooperated in the preceding round, all players assuredly cooperate in the following round. We call these strategies the cooperators and we calculate the robustness of these strategies by determining the proportion of cooperators that can resist invasion by all other strategies. We contrast this to the defectors: strategies which have $p^{0,0} = 0$, such that if al players defected in the preceding round, all players assuredly defect in the following round. The importance of these two strategy classes in two-player public goods games has been established already [3], making it natural to generalise their study to games with multiple players and long memory.

To determine whether a strategy is robust we use the condition given previously for an evolving population of N players in a multiplayer game. Given a resident strategy X in a population, selection acts against a new mutant Y provided $T^X(1) > T^Y(1)$, as described above. This can be written explicitly in terms of players' scores as

$$\frac{N-n}{N-1}S_0^X + \frac{n-1}{N-1}S_1^X > S_1^Y \tag{21}$$

where S_0^X is the score received by X with no mutants in the game, S_1^X is the score received by X with one mutant player Y in the game and S_1^Y is the score received by Y with no other mutants in the game.

5.2 Robust cooperating strategies under strong selection

We first identify the cooperating strategies that are robust under strong selection. As defined above, a cooperating strategy X is such that, if all players use the strategy, all players cooperate every turn at equilibrium. Such strategies must have $\kappa = B - C$.

A mutant strategy Y can selectively invade a cooperating strategy under strong selection iff

$$S_1^Y - S_1^X > \frac{N-n}{N-1}(B - C - S_1^X)$$

Thhe longterm payoffs must additionally satisfy Eq. 17-21. We can therefore identify strategies X which cannot be selectively invaded by any mutant Y. For simplicity we write

$$\hat{\Lambda}^{l_o,l_p} = \sum_{k=\max[0,l_o+l_p-(n-1)\times m]}^{\min[m,l_o]} \Lambda^{l_o+l_p-k,k} \frac{((n-1)\times m - l_o)!}{(m-k)!((n-2)\times m - l_o + k)!} \frac{l_o!}{k!(l_o-k)!} \frac{m!((n-2)\times m)!}{((n-1)\times m)!}. \tag{22}$$

Case I: Robustness when $\chi \leq \frac{N(n-2)+1}{(N-1)(n-1)}\phi$

Using Eq. 20 we can write the condition for invasion by a mutant strategy Y as

$$\left(\frac{N(n-2)+1}{(N-1)(n-1)}\phi - \chi\right)(S_1^X - (B-C)) < -\sum_{l_o=0}^{(n-1)\times m} \sum_{l_p=0}^{m} \hat{\Lambda}^{l_o,l_p} w^{l_o,l_p} \tag{23}$$

combining Eq. 16 with Eq. 19 then gives

$$\frac{N-n}{N-1} \sum_{l_o=0}^{(n-1)\times m} \sum_{l_p=0}^{m} \hat{\Lambda}^{l_o,l_p} w^{l_o,l_p} < C\left(\frac{N(n-2)+1}{(N-1)(n-1)}\phi - \chi\right) \sum_{l_o=0}^{(n-1)\times m} \sum_{l_p=0}^{m} C \frac{l_o}{(n-1)\times m} w^{l_o,l_p} \tag{24}$$

as a necessary condition for invasion.

Case II: Robustness when $\chi > \frac{N(n-2)+1}{(N-1)(n-1)}\phi$

Combining Eq. 16 and Eq. 20 we can also write

$$\frac{N}{N-1} \sum_{l_o=0}^{(n-1)\times m} \sum_{l_p=0}^{m} \hat{\Lambda}^{l_o,l_p} w^{l_o,l_p} < (B-C)\left(\frac{N(n-2)+1}{(N-1)(n-1)}\phi - \chi\right) \sum_{l_o=0}^{(n-1)\times m} \sum_{l_p=0}^{m} \frac{n\times m - l_o - l_p}{(n-1)\times m} w^{l_o,l_p} \tag{25}$$

as a necessary condition for robustness.

Thus, in summary, the set of robust cooperating strategies in an n-player game under strong selection with memory-m, which we denote $\mathcal{C}_s^{n,m}$, is given by:

$$\mathcal{C}_s^{n,m} = \Bigg\{ (\chi, \phi, \kappa, \Lambda^{0,1}, \dots \Lambda^{(n-1)\times m, m-1}) \Bigg| \kappa = B - C,$$
$$\frac{N-n}{N-1} \sum_{l_o=0}^{(n-1)\times m} \sum_{l_p=0}^{m} \hat{\Lambda}^{l_o,l_p} w^{l_o,l_p} \geq C \left(\phi \frac{N(n-2)+1}{(N-1)(n-1)} - \chi \right) \sum_{l_o=0}^{(n-1)\times m} \sum_{l_p=0}^{m} \frac{l_o + l_p}{(n-1)\times m} w^{l_o,l_p},$$
$$\frac{N}{N-1} \sum_{l_o=0}^{(n-1)\times m} \sum_{l_p=0}^{m} \hat{\Lambda}^{l_o,l_p} w^{l_o,l_p} \geq (B-C) \left(\phi \frac{N(n-2)+1}{(N-1)(n-1)} - \chi \right) \sum_{l_o=0}^{(n-1)\times m} \sum_{l_p=0}^{m} \frac{n \times m - l_o - l_p}{(n-1)\times m} w^{l_o,l_p} \Bigg\}$$
(26)

5.3 Robust defecting strategies under strong selection

We now identify the defecting strategies that are robust under strong selection. As defined above, a defecting strategy X is one such that, if all players adopt the strategy, all players defect every turn at equilibrium. Such strategies must have $\kappa = 0$.

A mutant strategy Y can selectively invade a defecting strategy under strong selection iff

$$S_1^X - S_1^Y < \frac{N-n}{N-1} S_1^X$$

Using Eq. 20 this can be re-written as

$$S_1^X \left(\frac{N(n-2)+1}{(N-1)(n-1)} \phi - \chi \right) < - \sum_{l_o=0}^{(n-1)\times m} \sum_{l_p=0}^{m} \hat{\Lambda}^{l_o,l_p} w^{l_o,l_p} \tag{27}$$

Following the same procedure as for the cooperators above, we find that the set of robust defecting strategies in an n-player game under strong selection with memory-m, which we denote $\mathcal{D}_s^{n,1}$, is given by

$$\mathcal{D}_s^{n,m} = \Bigg\{ (\chi, \phi, \kappa, \Lambda^{0,1}, \ldots \Lambda^{(n-1)\times m, m-1}) \Bigg| \kappa = 0,$$

$$\frac{N}{N-1} \sum_{l_o=0}^{(n-1)\times m} \sum_{l_p=0}^{m} \hat{\Lambda}^{l_o,l_p} w^{l_o,l_p} \geq -(B-C)\left(\phi \frac{N(n-2)+1}{(N-1)(n-1)} - \chi\right) \sum_{l_o=0}^{(n-1)\times m} \sum_{l_p=0}^{m} \frac{l_o + l_p}{(n-1)\times m} w^{l_o,l_p},$$

$$\frac{N-n}{N-1} \sum_{l_o=0}^{(n-1)\times m} \sum_{l_p=0}^{m} \hat{\Lambda}^{l_o,l_p} w^{l_o,l_p} \geq -C\left(\phi \frac{N(n-2)+1}{(N-1)(n-1)} - \chi\right) \sum_{l_o=0}^{(n-1)\times m} \sum_{l_p=0}^{m} \frac{n\times m - l_o - l_p}{(n-1)\times m} w^{l_o,l_p} \Bigg\} \tag{28}$$

Notice that in the special case $n = N$, in which all members of a population play the same public goods game together, the conditions for robust cooperation are independent of Λ^{l_o,l_p} and can never be satisfied.

5.4 Calculating robust volumes

We have now derived necessary and sufficient conditions for cooperators and defectors to be robust in n-player public goods games. However, in contrast to the case of two-player games these conditions depend explicitly on the equilibrium play w^{l_o,l_p} of an invading mutant. We can nonetheless easily construct strategies that are assuredly robust, by using the fact that $w^{l_o,l_p} \leq 1$ for all possible mutants. Similarly we can construct strategies that are assuredly invadable. However this leaves a large subset of strategies whose robustness depends on the actual values of w^{l_o,l_p}. Nonetheless we can still determine their robustness by using the fact that the bounds on players scores render the conditions Eqs. 26 and 27 most stringent when a mutant plays such that (1) he never cooperates at equilibrium (Eq. 16), (2) he plays so that $w^{0,0} = 0$ at equilibrium, i.e so that all players do not defect simultaneously (Eq. 18), (3) he always cooperates at equilibrium (Eq. 17) or (4) he plays such that all players do not cooperate simultaneously (Eq. 19). This leaves us with four possible trigger strategies to test in order to determine the robustness of a cooperator or defector strategy (where the relevant trigger strategy depends on the values of ϕ and χ and whether the resident is a cooperator or a defector).

Figure 3 of the main text verifies the use of these four trigger strategies by comparing the analytically predicted volumes of robust strategies to those estimated by Monte Carlo against a large number of randonly chosent mutant invaders. This type of Monte Carlo verification is also shown in Figure S1 for the effect of population size N on the volume of robust strategies. As discussed in the main text, larger

populations leader to larger volumes of robust cooperators and smaller volumes of robust defectors.

5.5 The impact of memory on robustness

As discussed in the main text, the impact of memory on robustness arises because it increases the capacity for contingent punishment, as expressed through the parameters Λ^{l_o,l_p}. The way in which this occurs is most clearly understood by looking at the expectation $\left\langle \Lambda^{l_o,l_p} \right\rangle$ for a randomly drawn strategy. For a randomly drawn cooperating or defecting strategy the expectations $\langle \chi \rangle$ and $\langle \phi \rangle$ are related according to

$$(n-1)\langle \chi \rangle = \tfrac{(n-1)}{C} - \langle \phi \rangle$$
$$1/2 = (B-C)(\langle \phi \rangle - \langle \chi \rangle)$$

which gives

$$\langle \chi \rangle = \tfrac{n-1}{Cn} - \tfrac{1}{2(B-C)n}$$
$$\langle \phi \rangle = \tfrac{n-1}{Cn} + \tfrac{n-1}{2(B-C)n}$$

The expectation $\left\langle \Lambda^{l_o,l_p} \right\rangle$ for a randomly drawn cooperator is then

$$\left\langle \Lambda^{l_o,l_p} \right\rangle = \frac{1}{2}\frac{l_o+l_p}{n \times m}$$

which gives an average across all l_o, l_p of

$$\langle \Lambda \rangle = \frac{1}{4}$$

Similarly, the expectation $\left\langle \Lambda^{l_o,l_p} \right\rangle$ for a randomly drawn defector is and the average Λ is

$$\left\langle \Lambda^{l_o,l_p} \right\rangle = -\frac{1}{2}\frac{n \times m - (l_o + l_p)}{n \times m}$$

which gives an average across all l_o, l_p of

$$\langle \Lambda \rangle = -\frac{1}{4}$$

If we now use Eq. 22 to determine the average $\left\langle \hat{\Lambda}^{l_o,l_p} \right\rangle$ for a cooperators and defectors faced with a mutant who cooperated l_p times within their memory, we recover Fig S2. We see that a randomly drawn cooperator tends to be more succesful at punishing a given mutant, while a randomly drawn defector tends to become less successful, as memory increases.

5.6 Invasability and cost of memory

Our evolutionary simulations, Figure 4, show that in addition to increasing the overall robustness of cooperation, memory capacity m tends to increase in small games. To understand this we must look at the average fixation probability of mutations that increase memory by 1, versus those that decrease memory by 1. This is shown in Figure S2c. We see that mutations that increase memory capacity are more likely to fix than mutations that decrease memory capacity, regardless of the current resident memory capacity. Thus longer memories will tend to evolve on average. If we introduce a cost for memory, so that a player's overall payoff is reduced by a factor mC_m, we see (Figure S2d) that mutations that increase memory eventually become worse invaders than mutations that decrease memory. In such cases an intermediate memory length evolves. Thus the evolution of memory depends on the costs associated with longer memories, as well as the size of the game being played. As we see in Figure S2a, shorter memories evolve, and much more slowly, when memory comes at a cost. Correspondingly (Figure S2b), the effect of evolving longer memories has a much weaker effect on the evolution of cooperation, although the general trend of reduced defection and increased cooperation is maintained.

5.7 Robustness and the dimension of strategy space

As discussed in the main text and shown in Figure 4, as memory increases the overall frequency of robust cooperators and defectors that evolve tends to decline. This reflects the fact that the absolute volume of

robust strategies tends to decline as the dimension of strategy space increases - the probability of randomly drawing a strategy from the n-dimensional unit cube, that also lies within a robust volume volume with sides of fixed length, declines as a power of $1/n$. This decline in the robust volumes of strategies with the dimension of strategy space (both game size n and memory length m) is shown in Figure S4.

6 Supplementary Figures

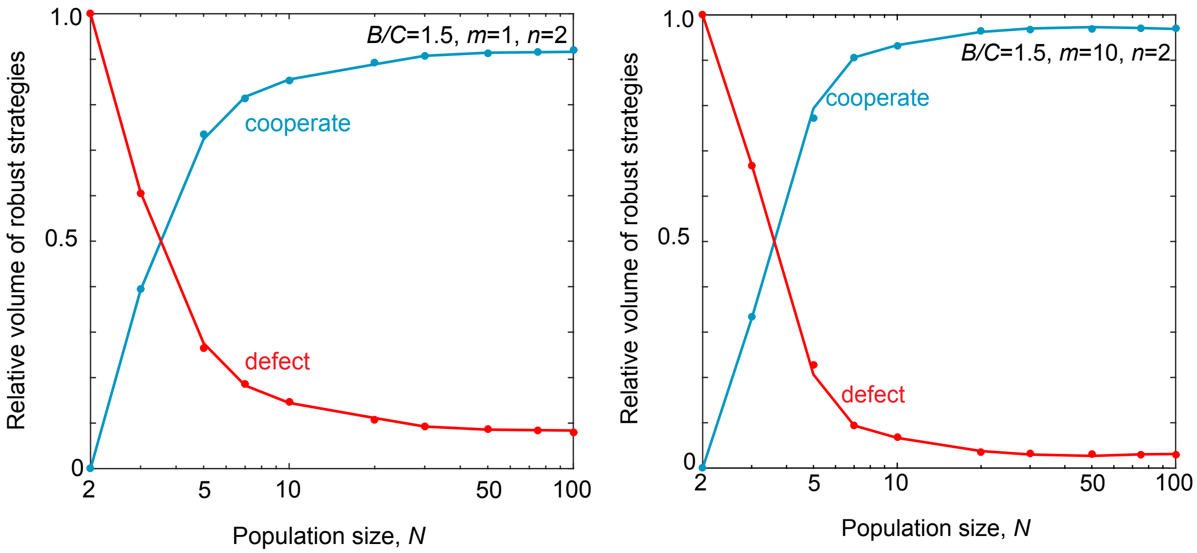

Figure S1: The impact of population size on cooperation. We calculated the relative volumes of robust cooperation – that is, the absolute volume of robust cooperative strategies divided by the total volume of robust cooperators and defectors – and compared this to the relative volume of defectors (solid lines) using Eqs. 2-3. We also verified these analytic results by randomly drawing 10^6 strategies and determining their success at resisting invasion from 10^5 random mutants (points). We calculated player's payoffs by simulating 2×10^3 rounds of a public-goods game. We then plotted the relative volumes of robust cooperators and robust defectors as a function of populations size N with fixed game size $n = 2$ and memory length $m = 1$, (left) and $m = 10$ (right). In both cases the effect of increasing population size is to increase the relative volume of cooperators and decrease that of defectors. In all calculations and simulations we used cost $C = 1$ and benefit B as indicated in the figure

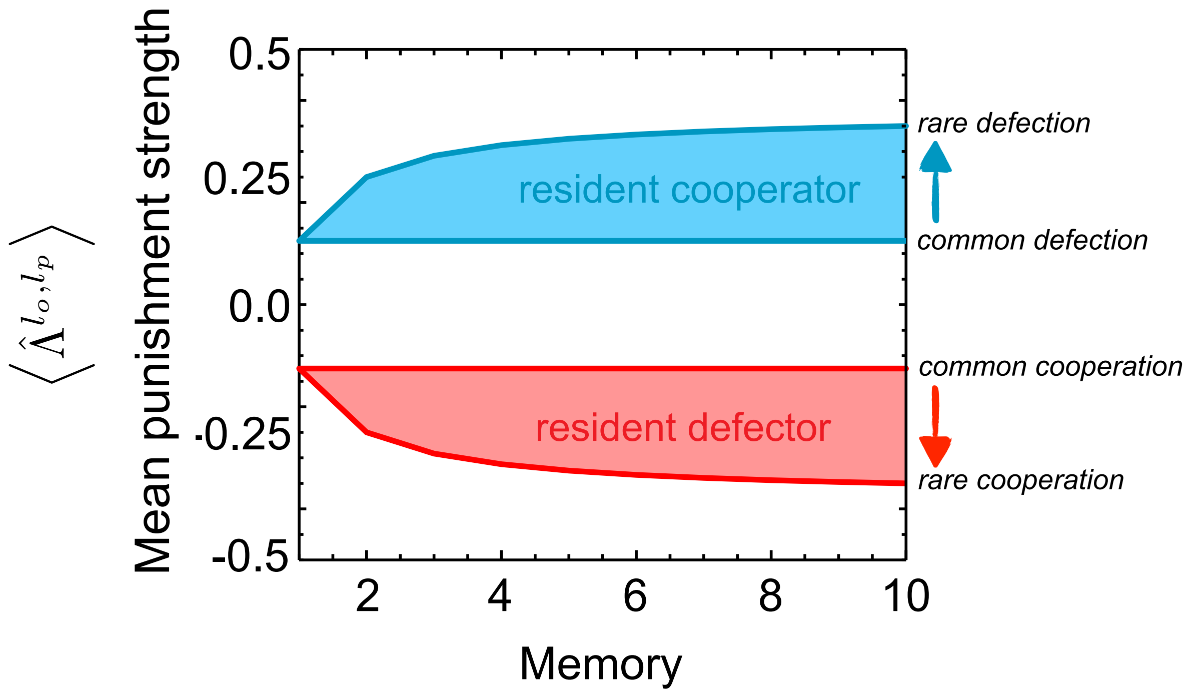

Figure S2: Effectiveness of contingent punishment. We calculated $\frac{1}{(n-1)\times m}\sum_{lo}\left\langle \hat{\Lambda}^{l_o,l_p} \right\rangle$ for the average punishment of a mutant who defected l_p times within the memory of the resident strategy, for both cooperators (blue) and defectors (red). As memory becomes longer, the average punishment increases for cooperators, making strategies more likely to be robust, and decreases for defectors, making strategies less likely to be robust.

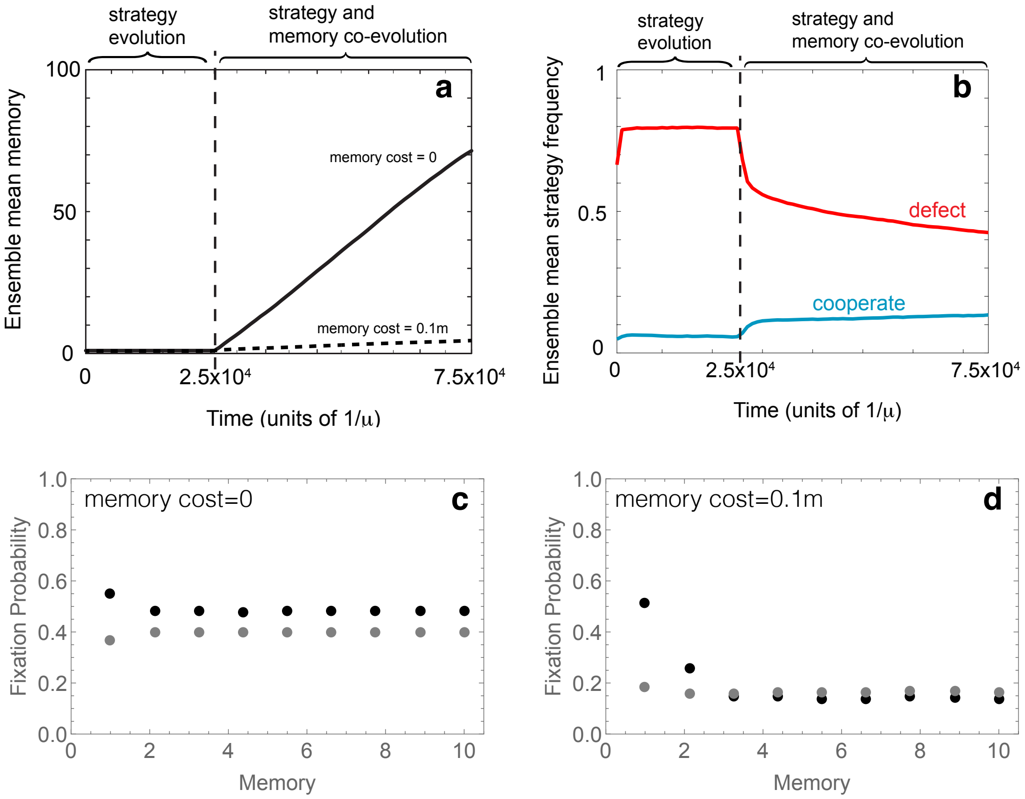

Figure S3: Invasibility of memory. We simulated co-evolution of memory and strategies as described in Figure 4 of the main text, with an additional cost to having memory which reduces a player's payoff by $c_m \times m$. We see that (a) much shorter memories evolve for $c_m = 0.1$ compared to $c_m = 0$ and (b) a correspondingly smaller amount of cooperation evolves. In order to understand why longer memory strategies evolve in small games we looked at the average fixation probability of mutations that increase or decrease memory, when played against a randomly drawn resident strategy. We drew 10^6 resident strategies for each memory length $m \in \{1, 2, 3, /ldots, 10\}$ and for each drew 10^5 mutants that increase memory length by 1 and 10^5 mutants that decrease memory length by 1. We assumed that a mutation that increased memory length by 1 did not change the probability p^{l_o,l_p} of the player's strategy. Where mutations increased memory length, we randomly drew probabilities $p^{(n-1)(m+1),l_p}$ and $p^{l_o,m+1}$. (c) Plotted are the average fixation probabilities for mutations that increase (black dots) or decrease (gray dots) memory by 1. Each point shows the probability of in versus out transition for the state k (i.e mutations that result in increase in memory from k to $k+1$ and mutations that result in decrease in memory from $k+1$ to k). When there is no cost to memory, mutations that increase memory length are always better invaders, for games of size $n = 2$. (d) When there is a cost to memory, mutations that decrease memory length do relatively better, and mutations that increase memory length do relatively worse. As a result we expect to see intermediate memory lengths evovle in the presence of costs.

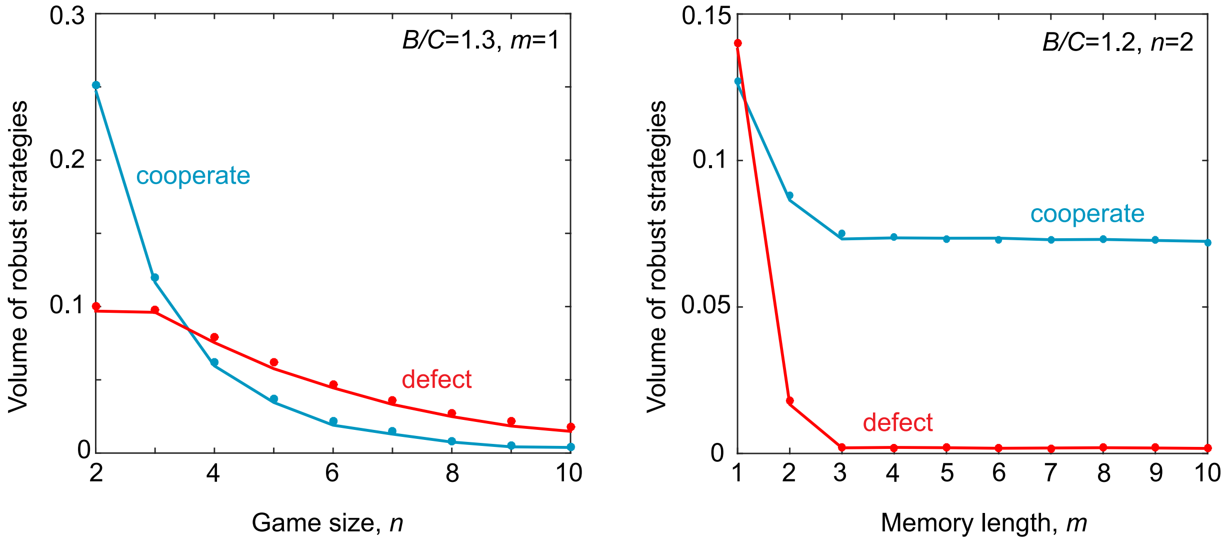

Figure S4: Absolute volumes of robust strategies. Here we show the same plot as in Figure 3 of the main text, using absolute rather than relative volumes. As is clear, the absolute volumes of both cooperators and defectors tends to decline as the dimension of strategy space increases. However this occurs at different rates for the different strategy types depending on whether game size (left) or memory (right) is increasing.

Literature Cited

[1] Press, W. H. & Dyson, F. J. Iterated prisoner's dilemma contains strategies that dominate any evolutionary opponent. *Proc Natl Acad Sci U S A* **109**, 10409–13 (2012).

[2] Akin, E. Stable cooperative solutions for the iterated prisoner's dilemma. *arXiv:1211.0969* (2012).

[3] Stewart, A. J. & Plotkin, J. B. Collapse of cooperation in evolving games. *Proc Natl Acad Sci U S A* **111**, 17558–63 (2014).

[4] Hilbe, C., Traulsen, A., Wu, B. & Nowak, M. A. Zero-determinant alliances in multiplayer social dilemmas. *arXiv:1404.2886v1* (2014).

[5] Pan, L., Hao, D., Rong, Z. & Zhou, T. Zero-determinant strategies in the iterated public goods game. *arXiv:1402.3542v1* (2014).

[6] Fudenberg, D. & Maskin, E. Evolution and cooperation in noisy repeated games. *American Economic Review* **80**, 274–279 (1990).

[7] Traulsen, A., Nowak, M. A. & Pacheco, J. M. Stochastic dynamics of invasion and fixation. *Phys Rev E Stat Nonlin Soft Matter Phys* **74**, 011909 (2006).

[8] Stewart, A. J. & Plotkin, J. B. From extortion to generosity, evolution in the iterated prisoner's dilemma. *Proc Natl Acad Sci U S A* **110**, 15348–53 (2013).

[9] Maynard Smith, J. The logic of animal conflict. *Nature* **246** (1973).

[10] Maynard Smith, J. *Evolution and the theory of games* (Cambridge University Press, Cambridge, 1982). URL `http://www.loc.gov/catdir/description/cam022/81021595.html`.
